# Establishment of dsDNA-dsDNA interactions by the condensin complex

**DOI:** 10.1016/j.molcel.2023.09.019

**Published:** 2023-10-10

**Authors:** Minzhe Tang, Georgii Pobegalov, Hideki Tanizawa, Zhuo A. Chen, Juri Rappsilber, Maxim Molodtsov, Ken-ichi Noma, Frank Uhlmann

**Affiliations:** 1 Chromosome Segregation Laboratory, The Francis Crick Institute, London NW1 1AT, UK; 2 Mechanobiology and Biophysics Laboratory, The Francis Crick Institute, London NW1 1AT, UK; 3 Department of Physics and Astronomy, University College London, London WC1E 6BT, UK; 4 Division of Genome Biology, Institute for Genetic Medicine, Hokkaido University, Sapporo, Hokkaido 060-0815, Japan; 5 Bioanalytics Unit, Institute of Biotechnology, Technische Universität Berlin, 13355 Berlin, Germany; 6 Wellcome Centre for Cell Biology, University of Edinburgh, Edinburgh EH9 3BF, UK; 7 Institute of Molecular Biology, University of Oregon, Eugene, OR 97403, USA; 8 Cell Biology Centre, Institute of Innovative Research, Tokyo Institute of Technology, Yokohama, Kanagawa 226-0026, Japan

## Abstract

Condensin is a structural maintenance of chromosomes (SMC) complex family member thought to build mitotic chromosomes by DNA loop extrusion. However, condensin variants unable to extrude loops, yet proficient in chromosome formation, were recently described. Here, we explore how condensin might alternatively build chromosomes. Using bulk biochemical and single-molecule experiments with purified fission yeast condensin, we observe that individual condensins sequentially and topologically entrap two double-stranded DNAs (dsDNAs). Condensin loading transitions through a state requiring DNA bending, as proposed for the related cohesin complex. While cohesin then favors the capture of a second single-stranded DNA (ssDNA), second dsDNA capture emerges as a defining feature of condensin. We provide complementary *in vivo* evidence for DNA-DNA capture in the form of condensin-dependent chromatin contacts within, as well as between, chromosomes. Our results support a “diffusion capture” model in which condensin acts in mitotic chromosome formation by sequential dsDNA-dsDNA capture.

## Graphical abstract

**Graphical abstract:**
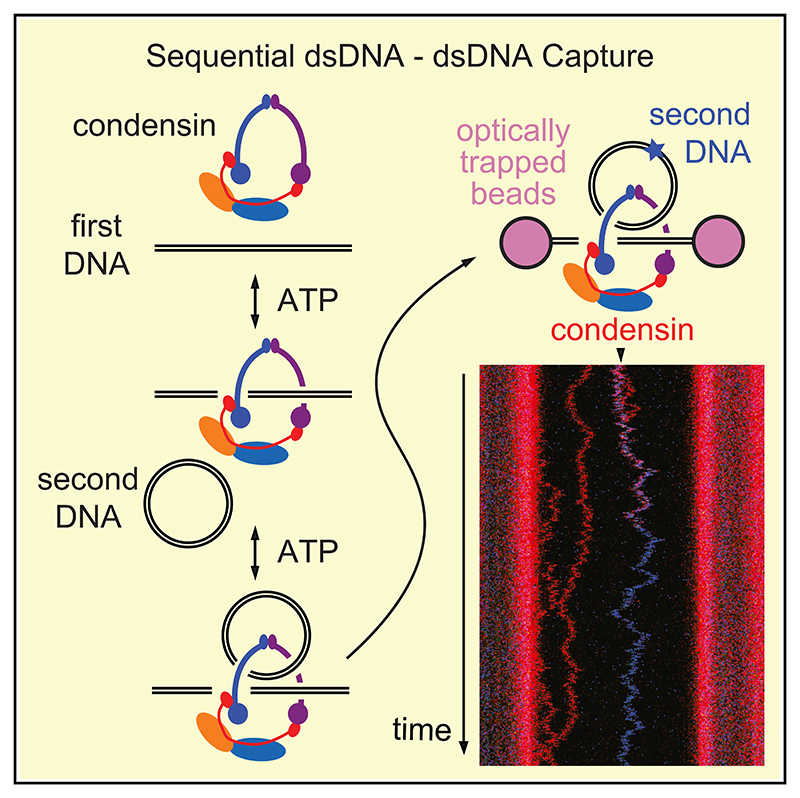

## Introduction

Genomic DNA undergoes a striking morphological transition as cells prepare for chromosome segregation during cell divisions. Chromatin that spreads throughout the interphase nucleus transforms into individual, rod-shaped mitotic chromosomes. A key player in chromosome formation is the ring-shaped condensin complex.^1,2^ Fission yeast cells lacking condensin fail to form mitotic chromosomes, resulting in fatal chromosome segregation failure.^3^ In higher eukaryotes, condensin gives chromosomes their shape and mechanical stability, whereas histone-histone contacts additionally contribute to mitotic chromatin compaction.^4–6^ Condensin acts by establishing long-range DNA contacts inside mitotic chromosomes,^7,8^ but how condensin forms these chromatin interactions has remained an unanswered question.

Condensins are molecular machines of the structural maintenance of chromosomes (SMC) family. They consist of two SMC subunits, Cut14^Smc2^ and Cut3^Smc4^, that dimerize at a “hinge,” from where coiled coils extend toward their ATPase heads. The heads are connected by a largely unstructured kleisin subunit, Cnd2^Brn1^, that completes the protein ring. Two HEAT (Huntingtin, Elongation factor 3, protein phosphatase 2A, and yeast kinase TOR1) repeat subunits, Cnd1^Ycs4^ and Cnd3^Ycg1^, further associate with the kleisin (fission yeast names are given, with those from other species in superscript, here from budding yeast).

A prominent model posits that condensin harnesses the energy from ATP binding and hydrolysis to actively extrude DNA loops.^9–11^ A series of loops then align to form a chromosome axis, where chromatin contacts arise. In this “loop extrusion” model, condensin naturally generates chromatin interactions within, but never between, chromosomes. The model is supported by *in vitro* single-molecule observations of loop extrusion on bare λ-DNA.^12^ Condensin can bypass individual DNA-bound obstacles during loop formation.^13^ However, loop extrusion is easily stopped by external force, and how condensin might navigate a complex *in vivo* chromatin landscape remains yet unknown.

A mechanistic understanding of loop extrusion is still being sought.^14–18^ Recently, contributions of individual condensin subunits were assigned. These studies revealed that vertebrate condensin lacking its Cnd3^CAP-G^ subunit is unable to extrude DNA loops but retains its ability to convert diffuse interphase chromatin into individualized and rod-shaped chromosomes, albeit somewhat wider than usual.^19,20^ These observations suggest that condensin can form chromosomes independently of loop extrusion.

Alternative to loop extrusion, condensin might establish long-range chromatin contacts by sequential capture of two neighboring binding sites when these meet by Brownian motion.^21,22^ In this “diffusion capture” model, condensin establishes interactions not only within, but at least occasionally also between, chromosomes. Computational diffusion capture simulations showed that contacts along the same chromatin chain have an advantage over interactions between chromosomes, over time resulting in chromosome compaction and individualization. The resultant simulated chromosomes display native-like features, e.g., local condensin clusters instead of the linear condensin backbone predicted by the loop extrusion model.^11,22,23^ A draw-back of the diffusion capture model is that it remains unknown whether, and if so how, condensin sequentially captures two DNA-binding sites. Furthermore, the feasibility of chromosome compaction by an indiscriminate capture mechanism in a real-life setting remains untested.

## Results

### Topological condensin loading onto DNA

**Figure 1:**
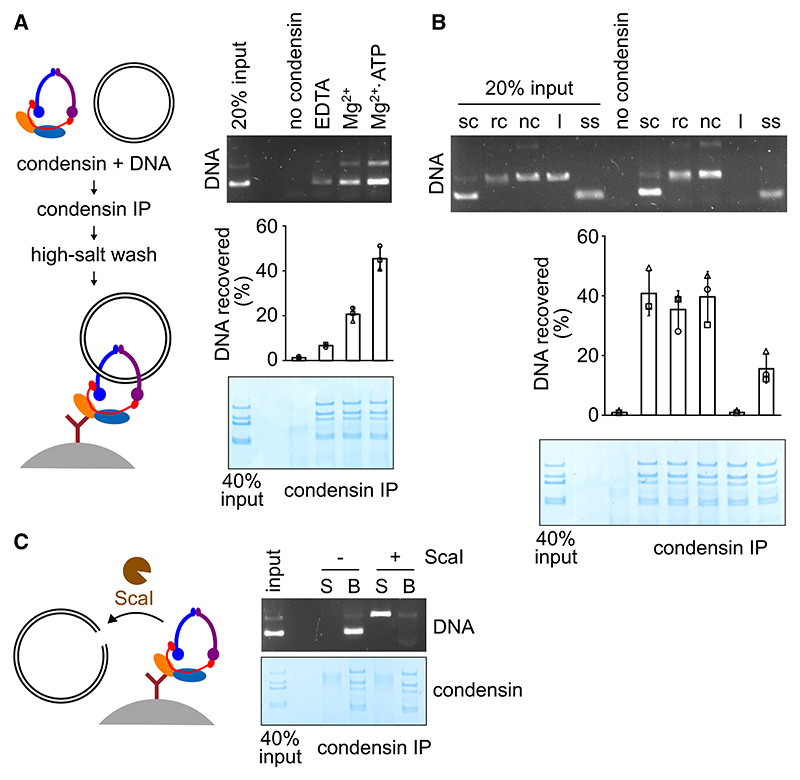
Topological condensin loading onto DNA (A) Schematic of the condensin loading assay. Agarose gel electrophoresis of the recovered DNA and its quantification relative to the input from three independent repeat experiments are shown. The bars and whiskers indicate means and standard deviations. Recovered condensin was analyzed by SDS-PAGE followed by Coomassie blue staining. (B) Condensin loading assay using DNA substrates of different topologies. sc, supercoiled circular dsDNA; rc, relaxed circular dsDNA; nc, nicked circular dsDNA; l, linearized dsDNA; ss, circular ssDNA. The bars and whiskers indicate means and standard deviations. (C) Schematic and result of a condensin loading assay followed by restriction enzyme cleavage of the circular dsDNA substrate. Recovered DNA and protein in the supernatant (S) and bead-bound (B) fractions are shown. See also Figure S1 for further biochemical characterization of fission yeast condensin.

We purified recombinant fission yeast condensin after co-overexpression of its five subunits in budding yeast (Figure S1A). Following incubation with circular pBlue-Script plasmid double-stranded DNA (dsDNA), we measured dsDNA binding following condensin immunoprecipitation and high salt (500 mM NaCl) washes. Over 40% of the input dsDNA was recovered in the presence of Mg^2+^ and ATP (Figure 1A). Without condensin, Mg^2+^, or ATP, no or only small amounts of dsDNA were retrieved. A reaction time course revealed that most dsDNA binding occurred within the first 15 min of incubation, whereas dsDNA binding by Walker A ATPase motif mutant condensin was not stimulated by ATP addition (Figures S1B and S1C). These results show that recombinant fission yeast condensin binds to DNA in an ATP-stimulated, high-salt-resistant manner, characteristic of topological DNA interactions by SMC complexes.

To investigate whether high-salt-resistant binding equates to topological DNA entrapment, we tested DNA substrates of different topologies. Three topologically closed dsDNA substrates—supercoiled, relaxed circular, and nicked circular—were all recovered with similar efficiency (Figure 1B). Condensin also bound, albeit less efficiently, circular single-stranded DNA (ssDNA). By contrast, we observed no detectable recovery of linear dsDNA, consistent with a topological condensin-DNA interaction. To further probe the condensin-DNA interaction, following circular dsDNA capture, we continued incubation in the absence or presence of the restriction endonuclease ScaI that cleaves the DNA substrate once (Figure 1C). Although an intact supercoiled plasmid remained stably bound to condensin in the bead fraction, linearized dsDNA was released into the supernatant. This experiment confirms that ATP-dependent condensin loading results in a topological DNA interaction.

Our findings so far support conclusions from two previous studies on topological DNA entrapment by budding yeast condensin. The first study^24^ demonstrated that condensin topologically entraps DNA *in vivo*, whereas the second^17^ suggested that following *in vitro* loading, DNA pseudo-topologically traverses condensin rings twice. The exact DNA path through condensin is inconsequential for our following considerations, although we note that the latter study employed a Cut14^Smc2^-Cnd2^Brn1^ fusion protein. If DNA usually enters the condensin ring through the Cut14^Smc2^-Cnd2^Brn1^ interface (the kleisin N-gate), akin to what was suggested for cohesin,^25^ the fusion will have inadvertently, but artefactually, generated the second observed DNA trajectory.

### Reversible loading and unloading

**Figure 2:**
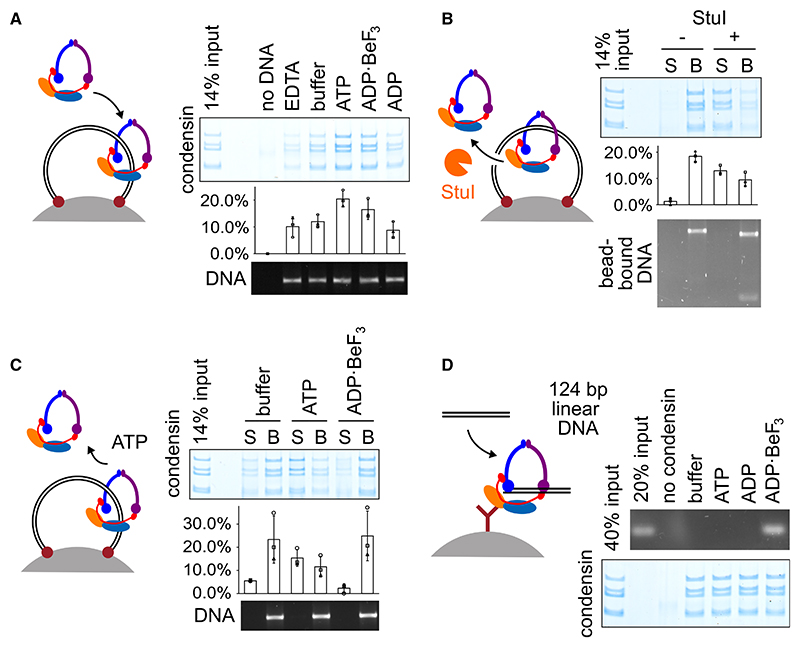
Reversible topological condensin loading and a condensin-DNA-gripping state (A) Schematic of the condensin loading assay using a bead-bound dsDNA substrate. SDS-PAGE analysis followed by Coomassie blue staining of recovered condensin and agarose gel electrophoresis of the bead-bound DNA are shown. Quantification of recovered condensin from three independent repeat experiments is displayed. Bars and whiskers indicate means and standard deviations. (B) The topological nature of condensin loading was probed by restriction enzyme cleavage of the bead-bound DNA. Recovered protein and DNA in the supernatant (S) and bead-bound (B) fractions were analyzed. Quantification of condensin in both fractions from three independent repeat experiments is shown. Bars and whiskers indicate means and standard deviations. (C) Reversibility of topological condensin loading. Following condensin loading onto bead-bound DNA, washed beads were further incubated in presence of the indicated nucleotides. Protein and DNA in the supernatant and bead-bound fractions were analyzed. Condensin quantification in both fractions from three independent repeat experiments is shown. Bars and whiskers indicate means and standard deviations. (D) Condensin-DNA-gripping state formed with a short linear dsDNA substrate in the presence of ADP·BeF_3_. See also Figure S2 for a condensin unloading time course and further characterization of the condensin-DNA gripping state.

Condensin binds chromosomes in a dynamic fashion.^19,26–28^ We therefore asked whether we could reconstitute not only condensin loading but also unloading from DNA. We modified our condensin loading assay and immobilized a linear 5 kb DNA, labeled with digoxigenin at both ends, onto α-digoxigenin antibody-coated beads. Following incubation with condensin, we observed ATP-dependent condensin retention (Figure 2A). Restriction enzyme cleavage confirmed the topological nature of the interaction (Figure 2B). Next, after washing away free condensin, we followed the behavior of DNA-bound condensin over the course of a second incubation. The majority of the DNA-bound condensin dissociated into the supernatant fraction if ATP was included in the second incubation, but not if ATP was omitted or replaced by the non-hydrolyzable ATP analog ADP ·BeF_3_ (Figure 2C). A time course experiment revealed that unloading was mostly complete within 15 min of the second incubation (Figure S2A). These results suggest that the five-subunit condensin complex contains all required activities to enact reversible ATP-dependent topological loading onto, as well as subsequent unloading from, DNA. This behavior differs from cohesin, which uses separate loading and unloading cofactors.^29^

### An ATP-bound condensin-DNA-gripping state

During loading, cohesin traverses an ATP-bound DNA-gripping state in which even short, linear DNA is tightly bound.^25^ To investigate whether condensin transitions through a similar loading intermediate, we incubated condensin with a 124 bp linear dsDNA substrate, followed by condensin immunoprecipitation. In the presence of ADP or ATP as the nucleotide, the linear dsDNA fragment was lost even following intermediate stringency washes (100 mM NaCl). By contrast, linear dsDNA was efficiently recovered in the presence of ADP ·BeF_3_ (Figure 2D). Eventually, after increasing the wash stringency to 500 mM NaCl, linear dsDNA was lost also in the ADP · BeF_3_ reaction (Figure S2B). DNA loss during the high-salt wash depended on the linear nature of the substrate. If condensin was bound to topologically constrained dsDNA in the presence of ADP · BeF_3_, the condensin-dsDNA interaction resisted high-salt washes (Figure 2A).

Thus, condensin, similarly to cohesin,^25^ transitions through an ATP-bound DNA-gripping state that is characterized by both electrostatic and topological DNA interactions. The biochemical similarities between the two SMC complexes open the possibility that cohesin and condensin load onto DNA in similar ways.

### Structure of the DNA-gripping state

**Figure 3:**
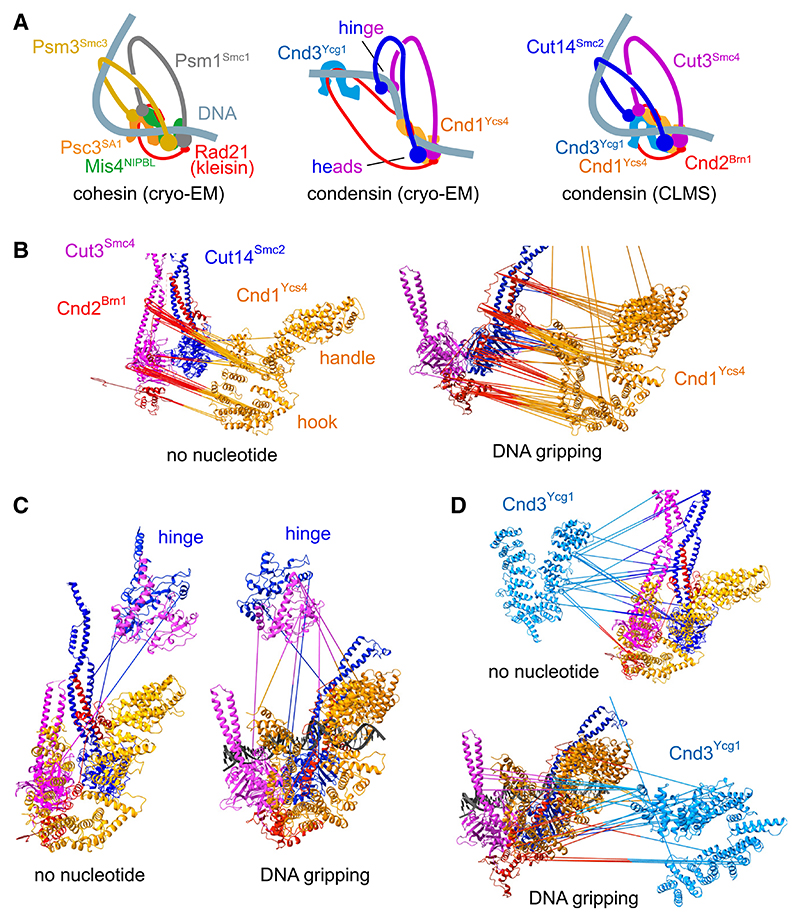
Architectural features of the condensin-DNA-gripping state (A) Schematics of cohesin^25,31,32^ and condensin^17,30^ in their ATP and DNA-bound states, seen by cryo-EM, as well as the condensin subunit arrangement derived from the CLMS experiment. (B) Protein crosslinks, detected by mass spectrometry, between Cnd1^Ycs4^ and the SMC subunits and kleisin Cnd2^Brn1^ in the no-nucleotide and DNA gripping states are shown, mapped onto structural models of fission yeast condensin in its respective states.^17,33^ (C) Crosslinks between the SMC hinge and a condensin structural core are shown. (D) Crosslinks between Cnd3^Ycg1^ and the condensin structural core. See also Figure S3 for additional documentation of the CLMS experiment.

Cryoelectron microscopy (cryo-EM) structures of budding yeast condensin in its ATP- and DNA-bound states^17,30^ have revealed not only similarities but also differences, compared with the cohesin-DNA-gripping state.^25,31,32^ Common to both states, one of the orthologous HEAT-repeat subunits (Cnd1^Ycs4^ in condensin and Mis4^NIPBL^ in cohesin) clamps DNA against the engaged SMC ATPase heads and the closed kleisin N-gate (Figure 3A). This arrangement provides the electrostatic as well as topological DNA interactions, which we have above biochemically confirmed as features of the fission yeast condensin gripping state.

In the case of cohesin, the two HEAT subunits stack up against each other, and the SMC hinge touches down to contact both.^25,31^ Condensin displays a different arrangement in the available cryo-EM structures,^17,30^ with the two HEAT subunits detached and the SMC hinge kinked only half-way toward the heads (Figure 3A). These distinctions could either point to mechanistic differences between condensin and cohesin or could have arisen due to technical reasons, e.g., HEAT-HEAT and HEAT-hinge interactions might have been lost during condensin cryo-EM sample preparation. To distinguish between the two possibilities, we mapped fission yeast condensin subunit interactions using protein crosslink mass spectrometry (CLMS).

We prepared two condensin-DNA samples, representing an initial state without nucleotide and the ADP · BeF_3_-bound DNA-gripping state (Figure S3A). We mapped the detected protein crosslinks onto structural models of fission yeast condensin, built using the respective budding yeast condensin structures in their nucleotide-free and DNA-gripping states^17,33,34^ as models. Crosslinks inside each subunit largely mapped within the 25 Å reach of the crosslinker (Figure S3B), validating our structural models and the CLMS experiment. We then turned to investigate crosslinks between subunits, comparing the no-nucleotide and gripping states (see the overview plots in Figure S3C).

The Cnd1^Ycs4^ subunit displayed expected and abundant crosslinks with the SMC heads and kleisin. In the initial state, these crosslinks were confined to the U-shaped Cnd1^Ycs4^ hook (Figure 3B). The gripping state contained numerous additional crosslinks between the Cnd1^Ycs4^ handle and both Cut14^Smc2^ and Cnd2^Brn1^ at the kleisin N-gate, which the HEAT subunit is known to keep shut at this stage.^17,30,33^ Our CLMS experiment thereby confirms prior structural knowledge about the behavior and conformational change of the Cnd1^Ycs4^ subunit during gripping state formation.

Looking at the SMC hinge, we made two observations. First, in the no-nucleotide state, we detected crosslinks between the hinge and the Cut3^Smc4^ and Cut14^Smc2^ coiled coils halfway toward the ATPase heads, recapitulating the conformation seen by cryo-EM^33^ (Figure S3D). Additionally, we detected a number of crosslinks between the SMC hinge and the ATPase heads and head-proximal coiled coil. These crosslinks indicate that the hinge, at least occasionally, reaches further down toward the heads (Figure 3C). We also observed hinge crosslinks with Cnd3^Ycg1^, suggesting conformational flexibility between these two subunits (Figures S3C and S3D). The number of hinge-head crosslinks increased in the gripping state when these contacts were joined by crosslinks between the hinge and both Cnd1^Ycs4^ and Cnd2^Brn1^. These additional crosslinks indicate a hinge interaction with the condensin structural core in the DNA-gripping state, analogously to what is seen in the cohesin-gripping state.^25,31^

Finally, we analyzed crosslinks of the second HEAT subunit, Cnd3^Ycg1^. In the initial state, most Cnd3^Ycg1^ crosslinks led to Cut14^Smc2^, close to where the subunit is seen in a DNA-free budding yeast structure.^33^ The Cnd3^Ycg1^ crosslink spectrum markedly changed in the gripping state. We now detected a series of crosslinks between Cnd3^Ycg1^ and Cnd1^Ycs4^, indicating that the two HEAT subunits interact (Figure 3D). The resulting placement of the condensin Cnd3^Ycg1^ subunit resembles the cohesin-gripping state,^25,31^ aligning closely with its HEAT partner Cnd1^Ycs4^. Taken together, our CLMS data show that fission yeast condensin, at least transiently, shares the subunit arrangements seen in the cohesin-gripping state (Figure 3A). These observations further support the possibility that both SMC complexes engage with DNA using a similar mechanism.

### Second dsDNA capture by condensin

**Figure 4:**
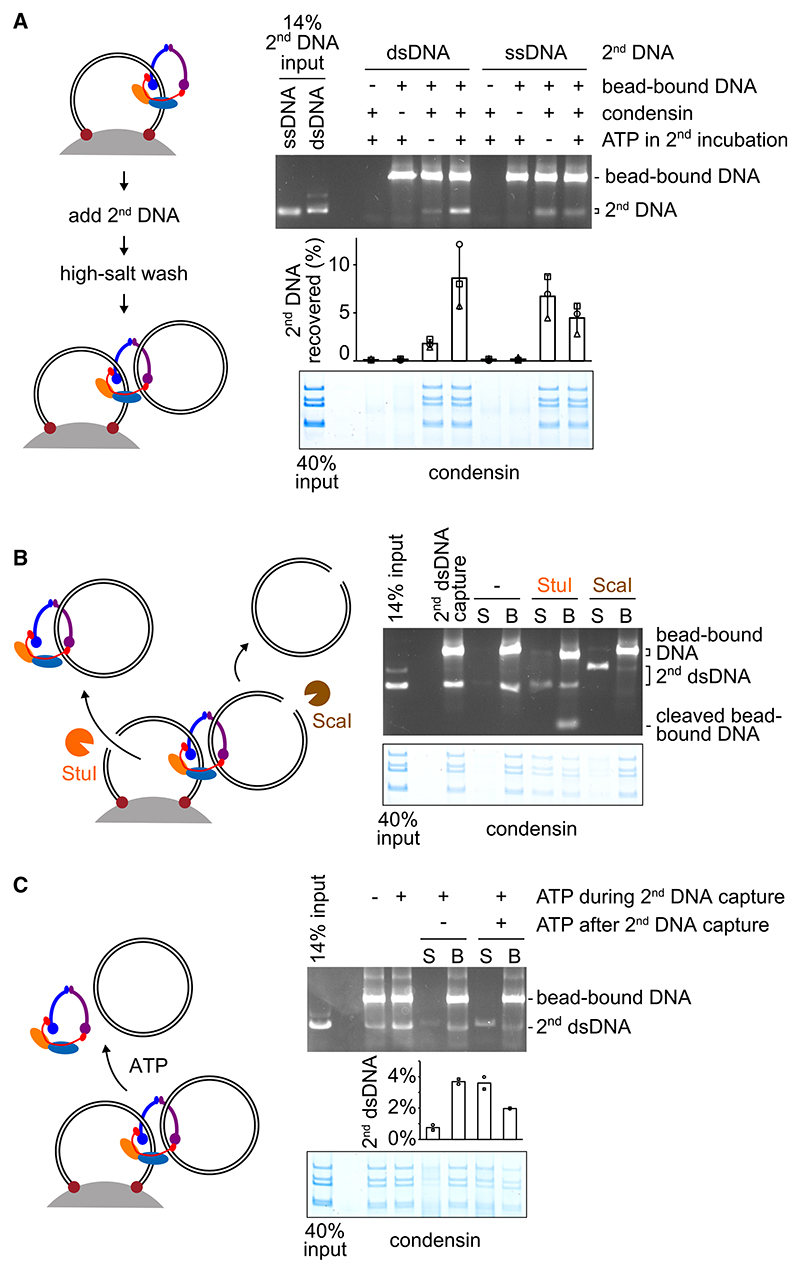
Second dsDNA capture by condensin (A) Schematic of the second DNA capture assay and agarose gel electrophoresis of recovered second ssDNA and dsDNA, as well as its quantification from three independent repeat experiments. The bars and whiskers indicate means and standard deviations. Recovered condensin was analyzed by SDS-PAGE followed by Coomassie blue staining. (B) The topological nature of second dsDNA capture was analyzed by restriction enzyme cleavage of either the bead-bound or the second DNA. (C) Reversibility of second dsDNA capture during a second incubation in the presence or absence of ATP. Condensin quantification in both supernatant and bead-bound fractions from two independent repeat experiments is shown, and the bar represents the mean. See also Figure S4 for additional characterization of second dsDNA capture by condensin.

After entrapping one DNA, cohesin captures a second DNA, preferentially a ssDNA.^35^ To investigate whether condensin is also capable of second DNA capture, we loaded condensin onto bead-bound dsDNA. After washing away unbound condensin, we added either double-or single-stranded circular plasmid DNA at a concentration equal to the amount of DNA present on the beads. Following incubation and washes, approximately 10% of the second dsDNA was captured when ATP was included in this reaction (Figure 4A). No second dsDNA was recovered in reactions without bead-bound DNA or condensin, whereas second dsDNA capture levels were markedly lower when ATP was omitted. ssDNA as the second substrate was also captured, albeit at lower levels. ATP addition reduced, rather than increased, second ssDNA recovery. These results suggest that condensin captures either a second dsDNA or ssDNA, with a preference for the former.

To probe the topological nature of second dsDNA capture, we repeated the capture experiment and then cleaved either the bead-bound or the second dsDNA with selective restriction enzymes, StuI and ScaI, respectively. Cleavage of the bead-bound DNA released a substantial portion of bead-bound condensin into the supernatant, accompanied by a similar fraction of second dsDNA (Figure 4B). Second dsDNA cleavage, in turn, resulted in dissociation of the linearized second dsDNA into the supernatant, whereas condensin remained bead-bound. These observations suggest that condensin’s interactions with both dsDNAs are topological in nature.

We also investigated the topological nature of second ssDNA capture. Cleavage of the bead-bound dsDNA again led to condensin and second ssDNA release (Figure S4A). To assess the topology of the ssDNA interaction, we used a second ssDNA substrate with four short, annealed oligonucleotide primers. Following the second ssDNA capture and washes, we converted the primed ssDNA to dsDNA using T4 DNA polymerase before testing the topological nature of the interaction by restriction enzyme cleavage (Figure S4B). Release following cleavage suggested that the second ssDNA was also topologically entrapped by condensin, with the caveat that we could only assess its topology following ssDNA to dsDNA conversion.

As condensin dynamically associates with DNA, we tested whether second DNA capture is reversible. Following a second dsDNA capture reaction and washes, we conducted further incubation. Without added ATP in the final incubation, both condensin and the second dsDNA largely remained a stable part of the bead-bound fraction (Figure 4C). By contrast, when ATP was included, over half of both condensin and the second dsDNA were released into the supernatant. These observations demonstrate that condensin establishes reversible topological interactions between two dsDNA substrates.

### DNA tension-restricted condensin loading and loop extrusion

Our above observations left open the question of whether individual condensins entrap two dsDNAs or whether more than one condensin cooperates in this activity. We therefore used single-molecule fluorescence microscopy to visualize the behavior of individual condensins. We fused the Cut3^Smc4^ C terminus to a CLIP tag, to which we coupled a Surface 647 dye during purification (Figure S5A). Bulk DNA loading and second DNA capture characteristics of the labeled condensin were indistinguishable from wild-type condensin (Figures S5B and S5C).

**Figure 5:**
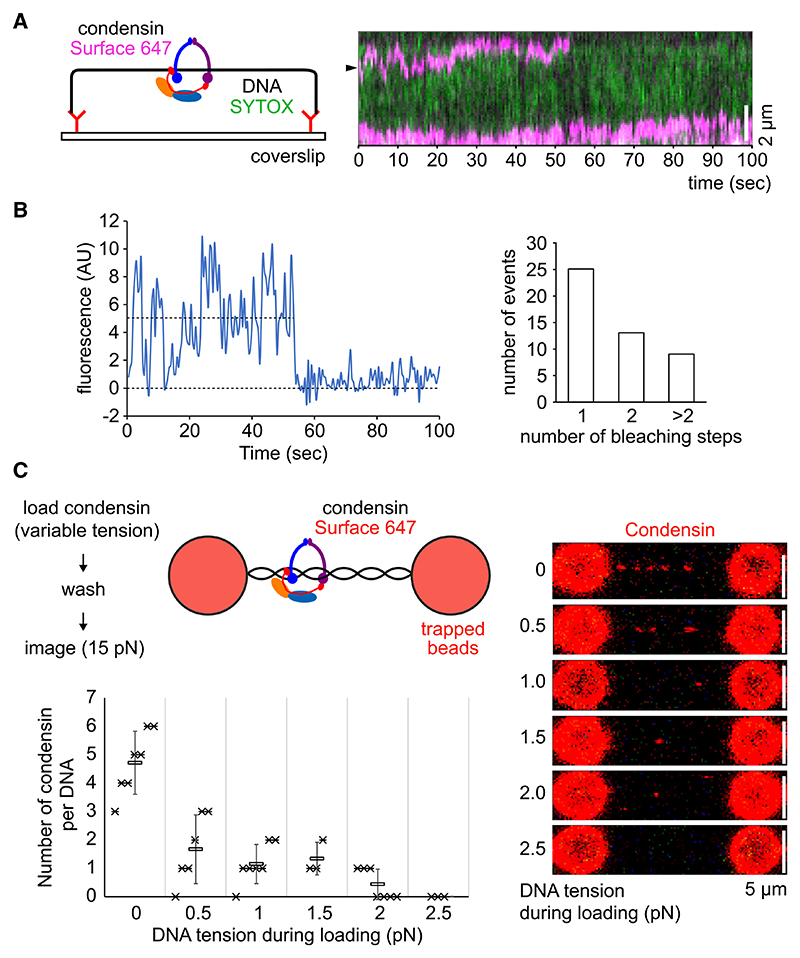
Tension-dependent single condensin loading onto DNA (A) Schematic of the digoxygenin-labeled *λ*-DNA, attached to an α-digoxygenin antibody-covered flow cell surface, as the substrate for single-molecule observation of Surface 647-labeled condensin loading by total internal reflection fluorescence (TIRF) microscopy. An example kymograph of a post-loading SYTOX Orange-stained DNA (green) with condensin (magenta) is shown. (B) Quantification of the Surface 647 intensity, highlighted by an arrowhead in the kymograph in (A), displaying single-step photobleaching. The distribution of condensin multiplicities on DNA, based on similar photobleaching curves, is shown. (C) Schematic and examples of DNAs incubated with condensin under the indicated stretch forces. The number of retained condensins was quantified. Each datapoint represents an independent experiment. The means and standard deviations at each force are shown. See also Figure S5 for additional characterization of the single-molecule imaging reagents, including confirmation of topological condensin loading onto *λ*-DNA and loop extrusion assays.

We now tethered 48.5 kb linear *λ*-DNA onto the coverslip surface of a microfluidic flow cell, then flowed in condensin and ATP, followed by a high-salt buffer wash. Surface 647-labeled condensin signals, most of which bleached in single steps suggestive of single condensins, linearly diffused along the *λ*-DNA (Figures 5A and 5B). Condensin could be pushed along the DNA by liquid flow and left the DNA following cleavage, as expected from a topological interaction (Figure S5D).

DNA entry into SMC rings through the kleisin N-gate involves DNA bending in the ATP-bound gripping state^25^ (compare Figure 3A). If indeed a DNA bend forms during loading, then applying tension to the DNA substrate should impede loading. To investigate this possibility, we used a C-Trap setup in which *λ*-DNA is suspended between two optically trapped beads (Figure 5C). We used this system to apply either no force to the *λ*-DNA or increasing stretch forces up to 2.5 pN before incubation with condensin. Following condensin exposure, we further stretched the DNA for washing in a high-salt buffer and imaging by confocal scanning fluorescence microscopy. This experiment revealed that condensin loaded efficiently onto relaxed DNA, but the loading efficiency rapidly declined with increasing tension. Condensin loading was barely observed when DNA tension exceeded 2 pN. The low force required to impede condensin loading suggests that largely spontaneous DNA bending forms part of the DNA entry reaction, consistent with the suggested bent DNA trajectory during kleisin N-gate entry.

Previous single-molecule experiments with condensin focused on characterizing its ability to extrude DNA loops on loosely tethered DNA substrates. We confirmed that our fission yeast condensin also possesses loop extrusion ability (Figure S5E). Looping efficiency and speed were comparable with that reported for budding yeast condensin.^12^ As in previous studies, efficient loop extrusion was observed in the presence of relatively high (250 nM) SYTOX Orange concentrations, an intercalating DNA dye that reduces both DNA diameter and persistence length.^36^ Thinner and more flexible DNA might promote the conversion of a bent DNA-loading intermediate into a loop.

To investigate the relationship between topological loading and loop extrusion, we asked whether loop-extruding condensins are topologically loaded, i.e., engage in a salt-resistant DNA interaction. Unlike topological DNA-DNA interactions,^35^ DNA loops formed by loop-extruding cohesins resolve when exposed to increased ionic strength.^37,38^ When we flowed in 300 mM NaCl-containing buffer following loop extrusion, condensin-made loops also resolved. In most observed cases (11 of the 15), condensin appeared to slide off DNA as loops resolved (Figure S5F). In other cases (4 of the 15), condensin remained DNA bound. These observations are consistent with the possibility that loop extrusion by condensin often occurs independent of, but in some cases co-exists with, topological loading.^18^ In other words, topological loading and loop extrusion are not mutually exclusive.

### Single condensins capture second dsDNA

**Figure 6:**
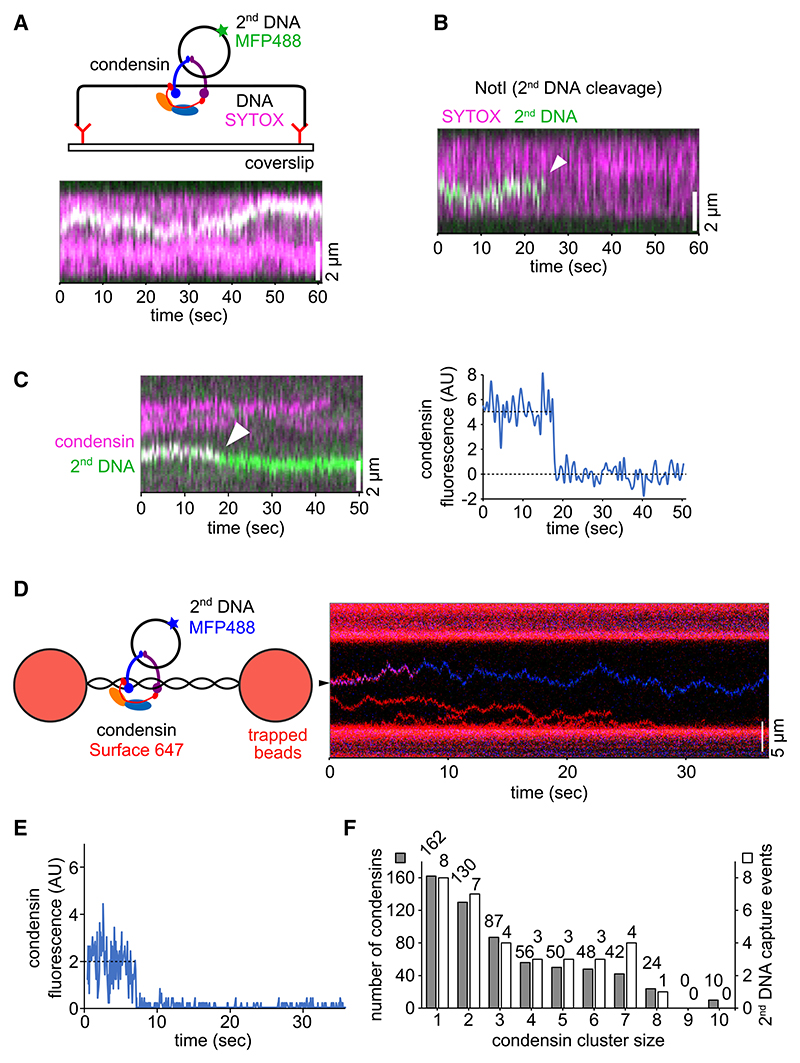
Second dsDNA capture by single condensins (A) Schematic and example kymograph of MFP488-labeled plasmid dsDNA (green) moving along a doubly tethered *λ*-DNA preincubated with condensin. After condensin loading and second DNA capture, DNA was stained with SYTOX Orange (magenta). (B) The topological nature of second dsDNA capture was probed by restriction enzyme NotI cleavage of the second dsDNA. The arrowhead highlights second dsDNA loss. (C) Surface 647-labeled condensin (magenta) was visualized together with the second dsDNA. Surface 647 fluorescence associated with the second dsDNA was recorded over time, revealing single-step photobleaching (highlighted by the arrow-head). (D) Schematic and example kymograph of second DNA (blue) capture by condensin (red) using the C-Trap setup. (E) Condensin fluorescence overlapping with the dsDNA signal, highlighted by an arrowhead in (D), was recorded over time, revealing single-step photobleaching. (F) The number of condensins in each observed fluorescent signal was counted, and then the cumulative number of condensins in signals of the indicated multiplicities was plotted (gray bars). The numbers of second DNA capture events associated with the same signals are plotted alongside (white bars). See also Figure S6 for quantification of condensin signals involved in second DNA capture in (C), a photobleaching trace of a signal containing two condensins from the experiment shown in (D), as well as real-time observation of second DNA capture by single condensins.

To address whether individual condensins can capture a second DNA, we chemically MFP488 fluorophore-labeled pBlueScript plasmid DNA that we flowed into a flow cell containing single condensins loaded onto tethered *λ*-DNA. Simultaneous imaging of the MFP488 label and *λ*-DNA showed the plasmid dsDNA diffusing along the *λ*-DNA (Figure 6A). No such events were seen if condensin was omitted from the experiment. Addition of the NotI restriction enzyme, which cleaves the plasmid once, resulted in plasmid signal loss in 6 of the 6 observed instances (Figure 6B), whereas the plasmid remained *λ*-DNA tethered during a control incubation (7 of the 7 observed events). These observations suggest that condensins topologically captured a second dsDNA in the flow cell.

Simultaneous imaging of Surface 647-labeled condensin and the MFP488-labeled DNA showed the plasmid colocalizing and co-diffusing with condensin along the *λ*-DNA in 20 of the 20 observed second DNA capture events (Figure 6C). Recording the condensin signal intensity over time showed single-step photobleaching in 5 of the 20 cases, suggesting that a single condensin trapped both the *λ*-DNA and the MFP488-labeled plasmid. In the other cases, more than one condensin colocalized with the second DNA (Figure S6A).

To observe second dsDNA capture by condensin in an independent manner, we again turned to the C-Trap. We sequentially incubated optically trapped *λ*-DNA with Surface 647-labeled condensin and the MFP488-labeled plasmid. The beads were then returned to the wash buffer channel for imaging. Although the *λ*-DNA itself remained invisible, we inferred its presence and integrity by the force response to bead pulling. Kymographs were then recorded by line scanning along the *λ*-DNA axis. These kymographs revealed labeled condensins diffusing along the *λ*-DNA, some of which moved together with an MFP488-labeled plasmid signal (Figure 6D), illustrating second dsDNA capture. We then measured photobleaching steps of all observed condensin signals, both those that captured a second dsDNA and those that did not (Figures 6E, 6F, and S6B). This analysis revealed that around a quarter of recorded second dsDNA capture events were mediated by a single condensin.

To further investigate whether single condensins are sufficient to capture a second DNA, we visualized condensin and DNA during the capture reaction. Despite the increased fluorescent background when imaging the second DNA-containing channel, we observed 19 instances where condensin caught a second DNA from solution. Six of these events were associated with condensin signals that bleached in a single step (Figure S6C). These observations further confirm that individual condensins can establish dsDNA-dsDNA interactions.

Although our experiments showed that single condensins can capture a second dsDNA, many second dsDNA capture events colocalized with signals of more than one condensin. We therefore evaluated whether the presence of multiple condensins increases the chance of second dsDNA capture. In the experiment shown in Figures 6D–6F, we counted 25 capture events among a total of 447 condensins contained in signals of more than one condensin. The resulting capture frequency per condensin of 5.6% was not statistically different from the 8 capture events recorded from 162 individual condensins, corresponding to a 4.9% capture frequency (*χ*^2^ proportions test) (Figure 6F). We do not know whether condensin signals with more than one condensin have a physiological meaning, but we can conclude that the ability to establish dsDNA-dsDNA interactions is a property of individual condensins.

### DNA-DNA capture by condensin *in vivo*

Our biochemical experiments showed condensin capturing two dsDNAs that must have found each other by Brownian motion. Whether condensin employs a similar diffusion capture mechanism to establish DNA interactions *in vivo* remains unknown. A key prediction from the diffusion capture mechanism is that condensin establishes DNA interactions not only within but, at least occasionally, between chromosomes. Loop extrusion, by contrast, should not result in condensin-mediated inter-chromosome contacts.

**Figure 7:**
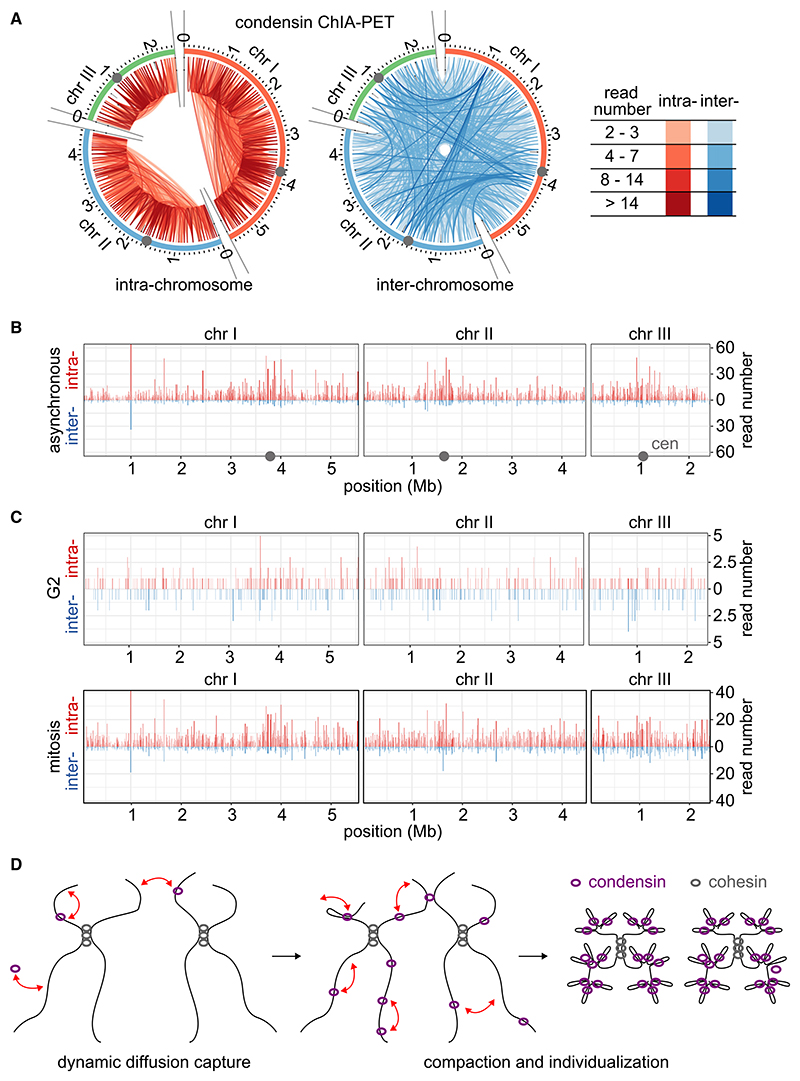
Condensin-linked intra- and inter-chromosome interactions (A) Circos plots of condensin-linked significant associations within (intra-chromosome) and between (inter-chromosome) the three fission yeast chromosomes. Color intensities reflect read number counts. Centromeres are represented by gray circles. (B) Intra- and inter-chromosome association counts were mapped along the three fission yeast chromosomes. (C) Intra- and inter-chromosome associations detected in G2- or mitosis-enriched fission yeast cultures are shown. (D) Model of mitotic chromosome formation by the diffusion capture mechanism. Intra-chromosome interactions have an intrinsic advantage and gradually replace inter-chromosome interactions, resulting in compaction and individualization of mitotic chromosomes. See also Figure S7 for additional documentation of the condensin ChIA-PET experiment as well as of the condensin-dependence of inter-chromosome interactions between condensin-enriched regions recorded by Hi-C.

To investigate whether condensin engages in inter-chromosome interactions, we examined chromatin interaction analysis by paired-end tag (ChIA-PET) sequencing datasets of condensin-linked chromatin contacts in fission yeast.^39^ Previously available analysis software employed a distance background model to determine significant associations, which limited the analysis to intra-chromosome associations. To overcome this limitation, we modified the Mango ChIA-PET analysis pipeline^40^ to include a background model for inter-chromosome interactions (see STAR Methods). We then defined significant associations as those with a false discovery rate (FDR) < 0.01 and a read count of at least two. This analysis revealed condensin-linked chromatin contacts both within as well as between the three fission yeast chromosomes (Figure 7A).

We next plotted the read counts of intra- and inter-chromosome associations as a function of genome coordinate. This depiction revealed a pattern of interaction hotspots along chromosome arms that was similar among intra- and inter-chromosome contacts (Figure 7B), although intra-chromosome interactions were represented by substantially larger read counts. This observation suggests that the same condensin-enriched regions engage in interactions both within chromosomes as well as between chromosomes.

In the diffusion capture model, the intrinsic advantage of polymer self-associations, compared with encounters between two polymers, gradually reinforces intra-chromosome interactions during chromosome formation.^21^ The above ChIA-PET data were obtained from asynchronously proliferating cells when most recorded associations stem from mitotic nuclei in which condensin is enriched.^39,41^ To address whether the condensin-linked chromatin interaction spectrum changes during mitotic chromosome formation, we compared ChIA-PET data obtained, following cell synchronization, from G2 and mitosis-enriched cell populations.^39^ Around 15% of cellular condensin is present in the interphase nucleus,^42^ which resulted in fewer detected chromatin associations. Despite their smaller numbers, these associations colocalized with interactions seen in the asynchronous and mitotic cultures (Figure S7A). Notably, G2 associations showed an almost equal distribution between intra- and inter-chromosome contacts (Figures 7C and S7B). By contrast, the mitotic cell population displayed markedly enriched intra-chromosome associations (p = 3.4 × 10^−12^, two-sided Fisher’s exact test). The observed shift from an equal level of intra- and inter-chromosome interactions to predominantly intra-chromosome interactions confirms predictions from the diffusion capture model.

A drawback of the ChIA-PET approach is that the method cannot ascertain whether observed condensin-linked contacts are indeed mediated by condensin. To address this question, we turned to Hi-C interaction data of mitotic fission yeast chromosomes, recorded in the presence or absence of condensin.^7^ The condensin-bound regions identified in our ChIA-PET analysis,^39^ as well as condensin-binding sites independently assigned by chromatin immunoprecipitation sequencing (ChIP-seq),^7^ were enriched for inter-chromosome interactions. This enrichment diminished following condensin depletion (Figure S7D), confirming that these inter-chromosome interactions were indeed mediated by condensin. Similarly, in budding yeast, prominent inter-chromosome interactions were reported between condensin binding sites at tRNA genes, which were lost upon condensin inactivation.^43,44^ Taken together, orthogonal approaches have detected condensin-mediated interactions not only within but also between chromosomes.

## Discussion

### Dynamic topological condensin loading and unloading

We reconstituted topological loading of the fission yeast condensin complex onto DNA *in vitro*, revealing biochemical characteristics similar to those observed with the related cohesin complex. A proposed DNA entry trajectory into the cohesin ring leads through the kleisin N-gate, which opens following ATP binding and shuts again after the DNA arrives.^25,45^ Structures of budding yeast condensin revealed a similar kleisin N-gate behavior: closed in an initial nucleotide-free state and open upon ATPase head engagement,^33,46^ only to be closed again after DNA arrival and gripping state formation.^17,30^ A defining feature of this entry trajectory is that DNA must bend to pass through the kleisin N-gate. We detected footprints of bent DNA in the cohesin-gripping state by DNA-protein crosslinking mass spectrometry,^25^ and we now present functional evidence that DNA bending forms part of the condensin loading reaction. Based on the accumulated evidence, we propose that DNA enters SMC rings by passage through the kleisin N-gate, a notion also supported by recent studies with the Smc5-Smc6 complex.^47^

By following the fate of condensin on DNA, we find that its five subunits are sufficient not only for loading but also for subsequent ATP hydrolysis-dependent unloading from DNA. Both reactions could therefore be functionally equivalent, i.e., loading through the kleisin N-gate could be naturally followed by unloading through the same gate. This possibility parallels observations with cohesin, where Pds5-Wapl catalyzes both DNA loading and unloading by opening the kleisin N-gate, at least *in vitro*.^29^ *In vivo* cohesin loading, by contrast, requires a specialized Mis4^Scc2^-Ssl3^Scc4^ cohesin loader complex. Although the Mis4^Scc2^ subunit is structurally similar to Pds5 and to condensin’s Cnd1^Ycs4^, Ssl3^Scc4^ is a unique addition. This additional subunit engages with chromatin receptors to target cohesin loading toward accessible DNA.^48,49^ Condensin makes use of its own chromatin receptors that recruit condensin to open chromatin regions, typically open gene promoters.^39,43,50–52^ How condensin engages with these receptors and whether this engagement involves Cnd1^Ycs4^ remains to be explored.

### Establishment of topological dsDNA-dsDNA interactions

Condensin’s role in chromosome formation requires that it establishes interactions between more than one DNA. We find that condensin sequentially and reversibly entraps two DNAs, preferentially two dsDNAs. This ability should allow condensin to pair any two of its binding sites and distinguishes condensin from cohesin, which favors the capture of a second ssDNA at DNA replication forks,^35^ and possibly elsewhere in the genome. What distinguishes condensin from cohesin when selecting a second DNA substrate will be important to explore. ssDNA is more flexible compared with dsDNA, which could give ssDNA an advantage if second DNA entry into cohesin was sterically more challenging. In the simplest scenario, the first and second DNAs follow the same trajectory through the kleisin N-gate into SMC rings. However, it remains to be explored whether this is indeed the case, as well as why this entry route might be sterically harder to navigate for a second time. Although dsDNA-dsDNA capture might be condensin’s defining feature, dsDNA-ssDNA interactions could also form part of its *in vivo* function. An ssDNA-binding protein mutation is an effective suppressor of a fission yeast condensin mutation.^53^

Although our experiments have established that individual condensins can capture two DNAs, protein interactions between condensin complexes could expand on such contacts to form association hubs. We have encountered assemblies of more than one condensin in our single-molecule experiments but do not currently know whether these assemblies are signs of higher-order structures or are merely coincidental. A physical interaction between vertebrate condensins has been reported,^20^ whereas bridging-induced phase separation offers another means by which more than one SMC complex might cooperate.^54^

### A loop-extrusion-independent model for chromosome formation

In the loop extrusion model for mitotic chromosome formation, ATP hydrolysis by condensin generates a weak ratchet force that causes DNA loops to slide over long distances.^9–12,15^ Whether these properties are commensurate with *in vivo* chromatin extrusion is unknown. In the alternative diffusion capture model, the energy for loop formation derives from Brownian motion, whereas ATP binding and hydrolysis merely control the capture mechanism.

A perceived drawback of diffusion capture is condensin’s inability to distinguish self from non-self. Instead of condensin, the continuity of the chromatin chain bestows an advantage to self-interactions. As chromosomes begin to individualize, self-interactions begin to dominate, whereas inter-chromosome interactions become less and less frequent (Figure 7D). The shifting interaction balance relies on the dynamic nature of the chromatin contacts that condensin establishes,^19,26–28^ which our experiments have biochemically confirmed. The challenge of chromosome formation grows in complexity with larger genomes. Higher eukaryotes make use of two distinct condensin complexes^1^ and they additionally rely on chromosome surfactants like Ki-67 to maintain chromosome individuality.^55^ Sequential topological entrapment of two DNA segments emerges as a powerful mechanism by which SMC complexes build chromatin interactions, not only those involved in sister chromatid cohesion and mitotic chromosome formation but also likely other genome transactions, including transcriptional regulation and DNA repair.

### Limitations of the study

Our topological condensin loading and second DNA capture assays were reconstituted using bare DNA substrates, whereas genomic DNA comes wrapped around histones and bound by additional chromatin-associated factors. Nucleosomes are known to restrict DNA access of the cohesin complex,^49^ and chromatin remodelers are required *in vivo* cofactors for both cohesin and condensin loading onto chromosomes.^51,56^ Indeed, in the diffusion capture model, the intervals in which condensin has access to the chromatin chain are an important determinant of the resultant chromosome architecture.^57^ The interplay between the chromatin landscape and chromosomal condensin loading sites is an important topic for further investigation.

The observation of sequential dsDNA-dsDNA capture by the condensin complex lends support to a diffusion capture model for mitotic chromosome formation.^22^ It will be important to delineate how diffusion capture co-exists with recently widely studied loop extrusion.^12^ Is condensin performing both activities during chromosome formation *in vivo*? Is loop extrusion an *in vitro* artefact of a bent DNA-loading intermediate turning into a loop, facilitated by DNA intercalating dyes? Our preliminary observations reveal that *in vitro* topological loading and loop extrusion are not mutually exclusive. Future experiments with SMC complexes that genetically separate their ability to topologically entrap DNA from their ability to extrude DNA loops will clarify how these two *in vitro* activities contribute to *in vivo* genome function.

## Star⋆Methods

### Key Resources Table

**Table:** 

REAGENT or RESOURCE	SOURCE	IDENTIFIER
Antibodies		
Mouse monoclonal anti-V5(Pk)	Bio-Rad	Cat# MCA1360; RRID:AB_322378
Mouse monoclonal anti-digoxygenin	Abcam	Cat# ab420; RRID:AB_304362
Chemicals, peptides, and recombinant proteins		
Rabbit IgG-Agarose	Merck	Cat# A2909
HiTrap Heparin HP 1ml	Cytiva	Cat# 17-0407-01
Superose 6 Increase 10/300 GL	Cytiva	Cat# 29-0915-96
cOmplete, EDTA-free protease inhibitor cocktail	Merck	Cat# 04693132001
M13KO7 helper phage	NEB	Cat# N0315S
Taq polymerase	QIAGEN	Cat# 201203
S400 spin column	Cytiva	Cat# 27514001
CLIP-Surface 647	NEB	Cat# S9234S
GelRed	Biotium	Cat# #41003-1
Polyethylenimine cellulose F sheets	Merck	Cat# 1055790001
Protein A dynabeads	ThermoFisher	Cat# 10002D
Protein G dynabeads	ThermoFisher	Cat# 10004D
Proteinase K	ThermoFisher	Cat# 25530049
IGEPAL	Merck	Cat# I3021-100ML
PMSF	Merck	Cat# 11359061001
4-12 % Tris-Glycine SDS-PAGE gels	ThermoFisher	Cat# XP04125BOX
γ-^33^P ATP	Hartmann	Cat# SRF-301-25
T4 DNA polymerase	NEB	Cat# M0203S
Bovin serum albumin (BSA)	Merck	Cat# A4503-500G
Fluidics tubing	Tygon	Cat# AAD04103
Pluoronic F-127	Anaspec	Cat# AS-84040
Digoxygenin-11-dUTP	Merck	Cat# 11093088910
G-50 spin column	Cytiva	Cat# 27533001
λ-DNA	NEB	Cat# N3011S
β-casein	Merck	Cat# C6905
syringe pump	Harvard Apparatus	Cat# Pico Plus Elite 11
SYTOX Orange	ThermoFisher	Cat#S11368
Taq DNA polymerase (for λ-DNA end filling)	NEB	Cat# M0273S
Biotin-16-dCTP	Jena Bioscience	Cat# NU-956-BIO14-S
Streptavidin-coated beads	Spherotech	Cat# SVP-40-5
Sulfo-SDA	ThermoFisher	Cat# 26173
Critical commercial assays		
NucleoSpin gel and PCR clean-up kit	Macherey-Nagel	Cat# 740609.250
Label IT nucleic acid labeling kit, MFP488	Mirus	Cat# MIR 7100
Deposited data		
Protein crosslinking mass spectrometry (CLMS) data	This study	JPST002102
Condensin ChIA-PET data	Kim et al.^39^	SRP061635
Unprocessed gel images	This study	https://doi.org/10.17632/gs8j93pct8.1
Experimental models: Organisms/strains		
Yeast strain to express fission yeast condensin	This study	Y5676
Antibodies		
Mouse monoclonal anti-V5(Pk)	Bio-Rad	Cat# MCA1360; RRID:AB_322378
Mouse monoclonal anti-digoxygenin	Abcam	Cat# ab420; RRID:AB_304362
Chemicals, peptides, and recombinant proteins		
Rabbit IgG-Agarose	Merck	Cat# A2909
HiTrap Heparin HP 1ml	Cytiva	Cat# 17-0407-01
Superose 6 Increase 10/300 GL	Cytiva	Cat# 29-0915-96
cOmplete, EDTA-free protease inhibitor cocktail	Merck	Cat# 04693132001
M13KO7 helper phage	NEB	Cat# N0315S
Taq polymerase	QIAGEN	Cat# 201203
S400 spin column	Cytiva	Cat# 27514001
CLIP-Surface 647	NEB	Cat# S9234S
GelRed	Biotium	Cat# #41003-1
Polyethylenimine cellulose F sheets	Merck	Cat# 1055790001
Protein A dynabeads	ThermoFisher	Cat# 10002D
Protein G dynabeads	ThermoFisher	Cat# 10004D
Proteinase K	ThermoFisher	Cat# 25530049
IGEPAL	Merck	Cat# I3021-100ML
PMSF	Merck	Cat# 11359061001
4-12 % Tris-Glycine SDS-PAGE gels	ThermoFisher	Cat# XP04125BOX
γ-^33^P ATP	Hartmann	Cat# SRF-301-25
T4 DNA polymerase	NEB	Cat# M0203S
Bovin serum albumin (BSA)	Merck	Cat# A4503-500G
Fluidics tubing	Tygon	Cat# AAD04103
Pluoronic F-127	Anaspec	Cat# AS-84040
Digoxygenin-11-dUTP	Merck	Cat# 11093088910
G-50 spin column	Cytiva	Cat# 27533001
λ-DNA	NEB	Cat# N3011S
β-casein	Merck	Cat# C6905
syringe pump	Harvard Apparatus	Cat# Pico Plus Elite 11
SYTOX Orange	ThermoFisher	Cat#S11368
Taq DNA polymerase (for λ-DNA end filling)	NEB	Cat# M0273S
Biotin-16-dCTP	Jena Bioscience	Cat# NU-956-BIO14-S
Streptavidin-coated beads	Spherotech	Cat# SVP-40-5
Sulfo-SDA	ThermoFisher	Cat# 26173
Critical commercial assays		
NucleoSpin gel and PCR clean-up kit	Macherey-Nagel	Cat# 740609.250
Label IT nucleic acid labeling kit, MFP488	Mirus	Cat# MIR 7100
Deposited data		
Protein crosslinking mass spectrometry (CLMS) data	This study	JPST002102
Condensin ChIA-PET data	Kim et al.^39^	SRP061635
Unprocessed gel images	This study	https://doi.org/10.17632/gs8j93pct8.1
Experimental models: Organisms/strains		
Yeast strain to express fission yeast condensin	This study	Y5676
Yeast strain to express fission yeast condensin carrying a Cut14-CLIP fusion	This study	Y5822
Yeast strain to express Walker A motif mutant fission yeast condensin	This study	Y6101
Software and algorithms		
Python script to analyse C-Trap data	This study	https://github.com/tonytang9544/CT6_C-Trap_kymograph_tracker

### Resource Availability

#### Lead contact

Further information and requests for resources and reagents should be directed to and will be fulfilled by the lead contact, Frank Uhlmann (frank.uhlmann@crick.ac.uk).

#### Materials availability

All unique reagents generated in this study will be made available upon reasonable request without restrictions.

### Experimental Model and Study Participant Details

The *S. cerevisiae* yeast strains used in this study for expression of the recombinant fission yeast *S. pombe* condensin complex were of the w303 background. Cells were cultured at 25°C.

**Table:** 

Strain number	Mating type	Genotype
Y5676	*MAT* **a**	*ade2-1, can1-100, ura3, psi^+^, pep4* *Δ* *::HIS3cnd2^+^-2xProtA-pGAL1-10-cut3^+^::LEU2* *GAL4-pGAL1-10-cut14^+^-3xPk::ADE2cnd1^+-^pGAL1-10-cnd3^+^::TRP1*
Y5822	*MAT* **a**	*ade2-1, can1-100, ura3, psi* *+* *, pep4* *Δ* *::HIS3cnd2^+^-2xProtA-pGAL1-10-cut3^+^::LEU2* *GAL4-pGAL1-10-cut14^+^-CLIP-3xPk::ADE2cnd1^+-^pGAL1-10-cnd3^+^::TRP1*
Y6101	*MAT* **a**	*ade2-1, can1-100, ura3, psi* *+* *, pep4* *Δ* *::HIS3cnd2^+^-2xProtA-pGAL1-10-cut3(K161I)::LEU2* *GAL4-pGAL1-10-cut14(K38I)-3xPk::ADE2cnd1 ^+-^pGAL1-10-cnd3^+^::TRP1*

### Method Details

#### Purification of recombinant fission yeast condensin

cDNA sequences of the *S. pombe* condensin complex subunits Cut14, Cut3, Cnd2, Cnd1, and Cnd3 were cloned into *S. cerevisiae* expression vectors under the control of the bidirectional *GAL1-10* promoter. The expression vectors were then integrated into a *pep4*D *S. cerevisiae* strain. The budding yeast were grown in YPR (Yeast Peptone medium with 2% Raffinose) at 25°C until OD_600_ reached between 1 and 1.5. 2% galactose was added to induce co-overexpression of the five condensin subunits for 4.5 hours before harvesting. Cells were washed with ice cold deionized water and resuspended in lysis buffer (40 mM Tris-HCl pH 7.5, 300 mM NaCl, 10% glycerol, 2 mM DTT, cOmplete protease inhibitor cocktail (Roche), 1 mM PMSF, 2 μg/ml RNase A) before flash freezing in liquid nitrogen. Cells were broken in a cryogenic freezer mill and the resulting powder was stored at -80°C until use. All remaining purification steps were performed at 4°C unless specified otherwise. The cell powder was thawed with an equal amount of lysis buffer and the extract was cleared by centrifugation at 105,000 x g for 45 minutes. The supernatant was then incubated with rabbit IgG-agarose beads (Sigma) for 2 hours. The beads were 6 times washed with 10 bead volumes of wash buffer (40 mM Tris-HCl pH 7.5, 300 mM NaCl, 10% glycerol, 2 mM DTT). The agarose beads were then resuspended overnight in wash buffer supplemented with 2 μg/ml RNase A and 20 μg/ml 3C protease. The eluted condensin was now applied to a Heparin column (Cytiva) equilibrated with Heparin buffer (20 mM Tris-HCl pH 7.5, 300 mM NaCl, 10% glycerol, 2 mM DTT). The column was washed with 20 column volumes of Heparin buffer before eluting in a linear gradient from 300 mM to 1 M NaCl in Heparin buffer. The peak fractions were pooled and concentrated by ultrafiltration before loading onto a Superose 6 column (Cytiva) equilibrated with gel filtration buffer (20 mM Tris-HCl pH 7.5, 200 mM NaCl, 10% glycerol, 2 mM DTT). Finally, the peak fractions were concentrated, aliquoted and flash frozen in liquid nitrogen for storage at -80°C. In the case of Cut14-CLIP-tagged condensin, the concentrated protein after the Heparin purification step was mixed with CLIP-Surface 647 (NEB) at a final concentration of 6 μM and incubated at 25°C for 4 hours before loading onto the Superose 6 column.

#### DNA substrates used in bulk biochemical assays

Supercoiled pBlueScript KSII (+) double stranded plasmid DNA was amplified in *E. coli* and purified by CsCl-gradient centrifugation. The purified DNA was adjusted to a concentration of 1 μg/μl in TE buffer (10 mM Tris-HCl pH 7.5, 1 mM EDTA). Relaxed circular, nicked circular, and linear pBlueScript dsDNA were generated by topoisomerase I, nicking enzyme and restriction enzyme treatment as described.^58^ pBlueScript single stranded DNA was generated using M13KO7 Helper Phage (NEB) according to the manufacturer’s instructions. The ssDNA was then purified by CsCl-gradient centrifugation as described.^35^

Four oligonucleotide primers were annealed to circular pBlueScript ssDNA to facilitate ssDNA to dsDNA conversion: 5‘-TCGCCA CTGGCAGCAGCCACTGGTAACAGGATTA-3’, 5’-CTAAATTGTAAGCGTTAATATTTTGTTAAAATTCGCGTTAAATTTTTGTTAAATCA GCTC-3’, 5’-GATGCTTTTCTGTGACTGGTGAGTACTCAACCAAGTCATTCTGAGAATAGTGTATGC-3’, and 5’-GCTTGATATCGA ATTCCTGCAGCCCGGGGGATCCACTAGTTCTAGAGCGGCCGCCACC-3’. 5 μg of pBlueScript ssDNA was mixed with 10 pmol of each primer in 40 μl 10 mM Tris-HCl pH 7.5, 50 mM NaCl, 1 mM EDTA. The mix was heated to 95°C for 1 minute and returned to 25°C at a rate of 1°C/minute. Excess primers were removed by passing through an S400 spin column (Cytiva) pre-equilibrated with 10 mM Tris-HCl pH 7.5, 0.1 mM EDTA.

A 124 bp linear dsDNA fragment to assemble the condensin-DNA gripping state was PCR amplified from pBlueScript dsDNA with primers 5’-CCGGCTCGTATGTTGTGTGG-3’ and 5’-GGTGGAGCTCCAGCTTTTGTTCC-3’ using Taq polymerase (Qiagen). The resulting PCR product was purified by TAE-agarose gel electrophoresis and the NucleoSpin Gel and PCR Clean-up kit (Macherey-Nagel).

Digoxygenin-labelled dsDNA (DIG-5kb-DIG), used as the bead-bound DNA substrate for condensin loading, unloading, and second DNA capture experiments, was synthesized by two-step PCR amplification. The first step used pEGFP-C1 (Clonetech) as the template and 5’- GGAAGCATAAAGTGTAAAGCCTGGGGCAAATATGTATCCGCTCATGAGACAATAACC-3’ and 5’- GCTTCCGGCT CGTATGTTGTGTGGAACCCTTTAGGGTTCCGATTTAGTGC-3’ as the primers. The PCR product was purified by TAE-agarose gel electrophoresis and served as the substrate for a second round of amplification using 5’-DigN/GCTAGGCATC/iDigN/GCTA GGCATC/iDigN/GCATAAAGTGTAAAGCCTGG-3’ and 5’-DigN/GCTAGGCATC/iDigN/GCTAGGCATC/iDigN/GGCTCGTATGTTGT GTGG-3’ as the primers. The PCR product was again purified by TAE-agarose gel electrophoresis.

#### Condensin loading assay using condensin immunoprecipitation

75 nM purified condensin was incubated with either 3.3 nM supercoiled pBlueScript dsDNA or 6.6 nM pBlueScript ssDNA as the substrate in 15 μl CL buffer at 30°C for 30 minutes. Then two 1.5 μl aliquots were retrieved to serve as the protein and DNA input controls, respectively. The remaining reaction was quenched by the addition of 0.2 ml cold CW buffer (40 mM Tris-HCl pH 7.5, 500 mM NaCl, 10% glycerol, 0.5 mM TCEP, 0.01% IGEPAL (Merck)). The mix was incubated with 10 ml protein A dynabeads (ThermoFisher), pre-adsorbed with 3 μg α-Pk antibody (BioRad), for 2 hours at 4°C. The beads were washed three times with 1 ml CW buffer and once with 1 ml CPE buffer (40 mM Tris-HCl pH 7.5, 100 mM NaCl, 10% glycerol, 0.5 mM TCEP, 0.01% IGEPAL, 5 mM MgCl_2_) before resuspension in 0.8 ml CPE buffer. 0.5 ml of the suspension was taken as the DNA sample (corresponding to 50% of the total reaction) and 0.25 ml was taken as the protein sample (corresponding to 25% of the total). To process DNA samples, the beads were collected and suspended in 10 μl DE buffer (35 mM Tris-HCl pH 7.5, 50 mM NaCl, 20 mM EDTA, 0.75% SDS, 2 mg/ml proteinase K (ThermoFisher)) and incubated at 50°C for 25 minutes. Eluted DNA was separated by agarose gel electrophoresis. In case of the protein samples, beads were collected suspended in 15 μl SDS-PAGE loading buffer and heated to 95°C for 5 minutes. Eluted protein was separated on 4-12% Tris-Glycine SDS-PAGE gels (ThermoFisher).

To evaluate the topological nature of the condensin-DNA interaction, beads containing condensin-DNA complexes, as above in 0.8 ml CPE, were collected and incubated in 10 μl NEB2.1 buffer (NEB, 50 mM Tris-HCl pH 7.5, 50 mM NaCl, 10 mM MgCl_2_, 0.1 mg/ml BSA) in the presence or absence of 10 Units ScaI-HF (NEB) at 18°C for 1 hour. Following the incubation, 10 μl NEB2.1 buffer adjusted to 500 mM NaCl was added and mixed vigorously. The supernatant was retrieved, and beads again resuspended in 20 μl NEB2.1 buffer. From both the supernatant and beads suspension, 12.5 μl (50% of the total reaction) and 7.5 μl (25% of the total) were processed as the DNA and protein samples, respectively, as described above.

#### Condensin gripping state formation

A 37.5 mM BeF_3_ stock was made freshly on the day of the experiment, by mixing 1 μl 0.1 M BeSO_4_,1 μl 0.5 M NaF, and 0.67 μl water, followed by room temperature incubation for 5 minutes. Condensin was then mixed with an equimolar concentration of 124 bp linear dsDNA in 15 μl CL buffer containing 0.5 mM of the indicated nucleotides at 30°C for 30 minutes. Two 1.5 μl aliquots were taken as the protein and DNA input samples. The remaining reaction was quenched by the addition of 0.2 ml cold CPE buffer. The mix was incubated with 10 μl Protein A dynabeads pre-adsorbed with 3 μg α-Pk antibody for 2 hours at 4°C. The beads were now washed three times with 1 ml CPE buffer, or for testing resistance to 500 mM NaCl with three times 1 ml CW buffer, then resuspended in 0.8 ml CPE buffer. The beads were split and processed as described for the condensin loading assay.

#### Condensin loading assay using bead-bound dsDNA

100 ng digoxygenin-labelled dsDNA (DIG-5kb-DIG) was incubated with 0.3 ng α-digoxygenin antibody (Abcam) in DBB buffer (40 mM Tris-HCl pH 7.5, 50 mM NaCl, 10% glycerol, 2 mM EDTA, 0.5 mg/ml BSA, 0.5 mM TCEP) at room temperature for 30 minutes. The mixture was then added to Protein G dynabeads (ThermoFisher) in PBSA-BSA buffer (5 mg/ml BSA dissolved in phosphate buffered saline) at 4°C for 2 hours. The beads were washed with CW buffer and equilibrated in CL buffer.

For a condensin loading and/or gripping assay, 150 nM condensin was added to the DNA beads in CL buffer, in the presence of the indicated nucleotides. The mixtures were incubated at 30°C for 30 minutes. Then the beads were collected and washed three times with 1 ml CW buffer, once with 1 ml CPE buffer, and then resuspended in 1 ml CPE buffer. From this suspension, 0.7 ml was taken as the protein sample (70% of the total reaction) and 0.25 ml was taken as the DNA sample (25% of the total). The beads in both aliquots were collected and processed as described above.

To investigate the topological nature of condensin binding to bead-bound DNA, beads were collected following the loading reaction and resuspended in 10 μl NEB2.1 buffer in the presence or absence of 10 units StuI (NEB) at 18°C for 1 hour. 10 μl NEB2.1 buffer containing 500 mM NaCl was added and vigorously mixed. The supernatant was retrieved, and beads were again resuspended in 20 μl NEB2.1 buffer. From both supernatant and bead suspension, 14 μl was taken as the DNA sample (70% of the total reaction) while 5 μl was taken as the protein sample (25% of the total). These samples were processed and analysed as described above.

To reconstitute condensin unloading, following condensin loading onto bead-bound DNA and washes as above, the beads were equilibrated in 15 μl CUL buffer (40 mM Tris-HCl pH 7.5, 135 mM KCl, 10% glycerol, 0.5 mM TCEP, 3 mM MgCl_2_, 0.1 mg/ml BSA) including the indicated nucleotides and incubated at 30°C for 30 minutes. The supernatants were retrieved, and the beads were resuspended in 15 μl CUL buffer. From both the supernatant and bead fractions, 10.5 μl were taken as the protein sample (70% of the total reaction) and 3.75 μl as the DNA sample (25% of the total). These samples were processed and analysed as described above.

#### Condensin second DNA capture assay

After topological condensin loading onto bead-bound dsDNA in the presence of ATP, as described above, the beads were washed 3 times with CPE buffer and once with CP2L buffer (40 mM Tris-HCl pH 7.5, 50 mM KCl, 10% glycerol, 0.5 mM TCEP, 3 mM MgCl_2_, 0.01% IGEPAL). The beads were then resuspended in 15 μl CL buffer containing 3.3 nM supercoiled pBlueScript dsDNA, or 6.6 nM pBlueScript ssDNA, as well as the indicated nucleotides, and incubated at 30°C for 30 minutes. The beads were washed 3 times with CW buffer, once with CPE buffer, and finally resuspended in 1 ml CPE buffer. 0.7 ml DNA sample (70% of the total reaction) and 0.25 ml protein sample (25% of the total) were processed and analysed as described above.

To study unloading of the second dsDNA, after second dsDNA capture and high salt washes, the beads were resuspended in 15 μl CUL buffer with the indicated nucleotide and incubated at 30°C for 30 minutes. The supernatants were retrieved, and the beads were resuspended again in 15 μl CUL buffer. From both the supernatant and the resuspended beads, 10.5 μl DNA sample (70% of the total reaction) and 3.75 ml protein sample (25% of the total) were processed and analysed as before.

To probe the topological nature of second dsDNA capture using restriction enzyme cleavage, after washes, the beads were resuspended in 10 μl NEB2.1 buffer in the presence or absence of 10 units StuI or 20 units ScaI-HF (NEB) at 18°C for 1 hour. 10 μl NEB2.1 buffer containing 500 mM NaCl was added and vigorously mixed. The supernatant was retrieved, and beads were again resuspended in 20 μl NEB2.1 buffer. From both supernatant and bead suspension, 14 μl was taken as the DNA sample (70% of the total reaction) while 5 μl was taken as the protein sample (25% of the total). The samples were processed and analysed as described above.

To investigate the topological nature of second ssDNA capture, pBlueScript ssDNA with 4 annealed primers was used as the substrate in the second ssDNA capture incubation. Following washes, the beads were resuspended in 10 μl T4 DNA polymerase buffer including 1.5 μl units T4 DNA polymerase (NEB) and 1 mM of each deoxynucleotide and incubated at 30°C for 30 minutes. The resultant dsDNA product was then subject to restriction enzyme treatment as above.

#### Single molecule fluorescence imaging

Hydrophobic coverslips were prepared as described.^59^ A glass cover slide was prepared by drilling holes to match the outer diameter of our Tygon AAD04103 fluidics tubing. The cover slide was cleaned by sequential sonication for 15 minutes in acetone, deionized water, and ethanol before air drying. A parafilm was placed on the cover slide from which 5 mm wide flow channels were excised. The hydrophobic coverslip was now positioned and fixed by brief heating of the parafilm on a 95°C heat block. Finally, plastic tubing was fixed to the cover slide using epoxy glue.

To prepare for imaging experiments, the flow cell was first equilibrated with BB buffer (50 mM Tris-HCl pH 7.5, 50 mM NaCl, 2 mM EDTA) and then incubated with BB buffer containing 10 μg/ml α-digoxygenin Fab fragment (Sigma) for at least 1 hour. The channel was washed with BB buffer and passivated with 1% Pluoronic F-127 (Anaspec) in BB buffer for 10 minutes. After additional washes, the channel was incubated with 1 mg/ml BSA in BB buffer for at least 3 hours before use.

0.5 mg λ-DNA (NEB) was labelled by filling the overhangs at both ends using Klenow fragment (NEB), dATP, dCTP, dGTP, and digoxygenin-11-dUTP (Merck) in the provided buffer in a 50 μl incubation. 2 μl 0.5 M EDTA pH 8.0 was added to quench the reaction before cleanup using a G-50 spin column (Cytiva) and stored at -20°C before use.

pBlueScript circular plasmid dsDNA was labelled with MFP488 fluorophores using the *Label* IT nucleic acid labelling kit (Mirus) according to the manufacturer’s instructions. 2 μl 0.5 M EDTA pH 8.0 was added to quench the 50 μl reaction mixture before cleanup using a G-50 spin column and storage at 4°C before use. Labelling efficiency was estimated from the absorbance spectrum of the labelled dsDNA product, comparing absorbance at 501 nm and 260 nm.

#### Single molecule condensin loading and second DNA capture assay

All experiments were performed at room temperature using a syringe pump (Harvard Apparatus) at a flow rate of 10 μl/min. The passivated flow channel was equilibrated thoroughly with BB buffer. Then 100 μl 0.2 ng/μl digoxygenin-labelled λ-DNA was introduced. The flow channel was washed again with BB buffer and then with BB buffer containing 5 nM SYTOX Orange to inspect the result of λ-DNA tethering. BB buffer containing 0.5 M NaCl was now applied to strip SYTOX Orange from the DNA. The channel was finally equilibrated again with BB buffer before subsequent experiments.

To observe condensin loading, the flow cell was equilibrated with TL buffer (40 mM Tris-HCl pH 7.5, 50 mM NaCl, 5% glycerol, 0.5 mM TCEP, 2 mM Trolox, 1 mg/ml BSA, 3 mM MgCl_2_, 1 mM ATP). Then 2 nM of Surface 647-labelled condensin in TL buffer was introduced into the flow channel for 3 minutes, followed by 3 minutes wash with TL buffer without MgCl_2_ and ATP. Finally, the flow channel was equilibrated with TW buffer (40 mM Tris-HCl pH 7.5, 300 mM NaCl, 5% glycerol, 0.5 mM TCEP, 2 mM Trolox, 1 mg/ml BSA, 100 nM SYTOX Orange) and imaged. 561 nm and 647 nm lasers were used to excite DNA-bound SYTOX Orange and Surface 647-labelled condensin, respectively.

To study the topology of the condensin-DNA interaction after loading, the flow cell was washed while imaging in TRE buffer (40 mM Tris-HCl pH 7.5, 100 mM NaCl, 5% glycerol, 0.5 mM TCEP, 2 mM Trolox, 1 mg/ml BSA, 10 mM MgCl_2_, 25 nM SYTOX Orange) in the presence or absence of 0.2 U/μl XhoI.

For condensin second dsDNA capture experiments, condensin was loaded onto surface-tethered λ-DNA in TL buffer as described above. The flow cell was washed with TL buffer without MgCl_2_ and ATP before 0.5 ng/μl of MFP488-labelled dsDNA plasmid in TL buffer was introduced for 5 minutes. Then the channel was washed with TW buffer and imaged. The MFP488-labelled dsDNA plasmid was visualized using a 488nm laser.

To investigate the topology of the condensin-second dsDNA interaction, positions of second DNA capture events were recorded. The flow cell was then rinsed while imaging in TRE buffer with or without 0.2 U/μl NotI for 15 minutes. After this treatment, the presence of the MFP488-labelled plasmid was examined at all recorded positions.

#### Single molecule image acquisition and analysis

A Nikon ECLIPSE Ti2-E TIRF microscope was used for image acquisition, equipped with a SR HP Apo TIRF 100x NA=1.49 WD=120 μm objective, using an angle between 58° to 60° to achieve highly inclined and laminated optical sheet (HILO) mode. Movies were recorded on NIS-Elements software (Nikon) using 16 bit HDR camera settings in the dual camera acquisition mode. The generated nd2 files were analysed using Fiji.^60^

To record photobleaching steps after condensin loading, its Surface 647 fluorescence signal was manually tracked using a circular region of interest. After photobleaching, the region was held at the last visited location. Background fluorescence was defined as the mean fluorescence in the region after photobleaching and was subtracted from all frames.

To count the number of condensin photobleaching steps after second DNA capture, the MFP488 signal from the second dsDNA was used for tracking using the “TrackMate” plugin^61^ in Fiji. The fluorescence intensity in the condensin channel was then extracted from the track, plotted against time, and the number of photobleaching steps was counted.

#### DNA stretch force-dependent condensin loading

λ-DNA was biotinylated at both ends by filling its overhangs. A 50 uL reaction contained 12.5 μg λ-DNA, 0.5 μl Taq DNA polymerase (NEB), and 10 μM of dATP, dTTP, dGTP (Promega), as well as Biotin-16-dCTP (Jena Bioscience), in Taq buffer (NEB). Incubation was for 30 minutes at 72°C. DNA was separated from unincorporated nucleotides using a Micro Bio-Spin P30 gel-filtration column (Bio-Rad).

The following experiments were performed at room temperature using a C-Trap optical trap system (Lumicks). Two streptavidin-coated beads of 4.8 mm diameter (Spherotech) were trapped in the beads channel by two laser beams. The beads were then moved to a channel containing 3 pM biotinylated λ-DNA for DNA capture. Attachment of a single λ-DNA was confirmed by comparing the force-extension curve against those prerecorded in the C-Trap operating system.

To assess the DNA stretch force-dependence of condensin loading, the λ-DNA was relaxed to a 5 mm end-to-end distance, corresponding to 0 pN tension. The λ-DNA was then moved, or stretched with the indicated force before moving, to a channel containing 2 nM Surface 647-labelled condensin in CTL buffer (50 mM Tris-HCl pH 7.5, 20 mM NaCl, 3 mM MgCl_2_, 1 mM ATP, 1 mg/ml β-casein) for one minute. The stretching force was increased to 15 pN in constant force mode and, after locking the trap in constant distance mode, the stretched λ-DNA was moved to the imaging channel containing CTI buffer (50 mM Tris-HCl pH 7.5, 300 mM NaCl, 3 mM MgCl_2_, 1 mM ATP, 1 mg/ml β-casein) and imaged by confocal fluorescence microscopy with excitation from the 647 nm laser at 10% of its maximum power. A movie of an area encompassing the two beads and intervening space was recorded with a pixel size of 100 nm and 100 μs exposure per pixel.

For condensin second DNA capture, relaxed λ-DNA was incubated for 1 minute in the condensin channel containing 2 nM Surface 647-labelled condensin in CTL buffer. The λ-DNA was then stretched to 15 pN using constant force mode and moved to the second DNA capture channel containing 0.8 ng/μl MFP488-labelled pBlueScript in CTL buffer for 1 minute. After switching to constant distance mode, the stretched λ-DNA was moved to the imaging channel containing CTI buffer and imaged using confocal fluorescence microscopy with excitation from the 488 nm and 647 nm lasers both at 10% of their maximum power. To achieve higher time resolution, a 1-pixel wide line scan movie along the tethered λ-DNA was recorded.

#### Image analyses of C-Trap data files

The h5 files containing the scan movies were exported to tiff format for visualization in Fiji. To determine the number of topologically loaded condensins under varying DNA tension, the condensin fluorescence signal was extracted from a thin rectangular region of interest (ROI) between the two streptavidin beads. The background fluorescence signal was determined from an area of the same shape, next to the ROI. The average background fluorescence was subtracted from each pixel in the ROI, followed by 5x5 binning, resulting in bins of the approximate dimensions of the observed condensin signals. Fluorescence intensities of the binned data was plotted as a histogram from which the single condensin intensity becomes apparent. The total number of loaded condensins was then calculated by dividing the total fluorescence signal in the ROI by the signal from a single condensin. In each case, the condensin count based on total intensity was confirmed by counting individually visible and mobile signals in the original movie.

To evaluate the number of condensins that participate in second DNA capture, we generated a custom Python script (https://github.com/tonytang9544/CT6_C-Trap_kymograph_tracker) that used the “track_greedy” function in Pylake (Lumicks) to track the diffusion-limited second DNA MFP488 signals on kymographs. Then the Surface 647 intensity, corresponding to condensin, was extracted from these same areas, and plotted over time. Photobleaching steps were determined from these traces and the number of condensins was determined by dividing the initial Surface 647 intensity by the intensity loss during the photobleaching step(s).

#### DNA loop extrusion assay

Microfluidic flow cells were prepared as previously described^15^ and incubated with 1 μl of α-digoxigenin antibody (Roche) diluted in 30 μl of TB buffer (40 mM Tris-HCl pH 7.5, 50 mM NaCl) for 10 minutes, followed by a wash with 400 μl TB. The surface was passivated by sequential incubation with 50 μl of 1% Pluronic F127 (Sigma-Aldrich) in TB for 10 minutes, followed by a 400 μl TB wash and incubation with 30 μl of 10 mg/mL β-Casein (Sigma-Aldrich) in TB for 30 minutes, and by a final wash with 400 μl of TB. 40 μl of 15 pM λ-DNA (NEB) in TB, digoxigenin-labelled at both ends,^15^ was introduced into the flow cell at a flow rate of 4 μl/min. DNA remained in the flow cell for 10 minutes followed by a 40 μl TB wash at the original flow rate of 10 μl/min.

The flow cell was now equilibrated with 50 μl buffer R (40 mM Tris-HCl pH 7.5, 50 mM NaCl, 2 mM MgCl_2_, 5 mM ATP, 10 mM DTT, 250 nM SYTOX Orange, 0.2 mg/mL glucose oxidase, 35 μg/mL catalase, 4.5 mg/mL glucose and 0.1 mg/mL β-Casein) at a flow rate of 15 μl/min. 5 nM condensin was introduced into the flow cell in buffer R at 10 μl/min. DNA molecules stained with SYTOX Orange were imaged using a custom-built highly inclined laminated optical sheet (HILO) microscope, utilizing a 561 nm laser and a Nikon SR HP Apo TIRF 100x/1.49 oil immersion objective at 1 Hz acquisition frequency and 100 ms exposure times. Images were collected with an Andor Sona sCMOS camera, saved as uncompressed TIFF files and further processed using ImageJ.

For experiments visualizing both condensin and DNA, 0.5 nM Surface 647-labelled condensin was introduced into the flow cell in buffer R at 10 μl/min. Alternating excitation between 561 nm and 647 nm lasers was used for visualization of DNA and condensin. Images were collected using two Andor Sona sCMOS cameras at 1 Hz acquisition frequency, 100 ms exposure, and 2x2 binning.

To determine loop extrusion rates, the lengths of DNA molecules undergoing loop extrusion was manually measured over time using ImageJ. To convert distance in pixels into kbp, the length of each DNA molecule before loop initiation was measured, averaged over a 5-second interval, and normalized to a total DNA length of 48.5 kbp. Loop extrusion of each DNA molecule was analysed frame by frame by measuring the length of the remaining DNA outside of the loop and subtracting it from the total DNA length prior to loop initiation. The rate of DNA loop extrusion was then calculated as the slope of a linear fit to the experimental data.

To investigate the topology of the condensin-DNA interaction following loop extrusion, we first equilibrated a flow cell with 50 μl buffer RX (40 mM Tris-HCl pH 7.5, 2 mM MgCl_2_, 0.5 mM TCEP, 200 nM SYTOX Orange, 2 mM Trolox, 0.2 mg/mL glucose oxidase, 35 μg/mL catalase, 4.5 mg/mL glucose and 1 mg/mL β-Casein) supplemented with 50 μM NaCl and 5 mM ATP at a flow rate of 15 μl/min. Next, 50 μl of 6 nM Surface 647-labelled condensin was introduced into the flow cell in buffer RX supplemented with 50 mM NaCl and 5 mM ATP at 10 μl/min. To remove free condensin and ATP, the flow cell was then washed with 50 ml buffer RX supplemented with 50 mM NaCl at 10 μl/min. Finally, the flow cell was transfused with 50 ml buffer RX supplemented with 300 mM NaCl, while imaging using alternating excitation between 561 nm and 647 nm lasers at 0.5 Hz and 100 ms exposure, to observe DNA and condensin behavior during loop resolution.

#### Crosslink mass spectrometry (CLMS) sample preparation

50 μg condensin was mixed with an equimolar concentration of a 124 bp linear dsDNA fragment in the presence or absence of 0.5 mM ADP**·**BeF_3_ in CL buffer and incubated at 30°C for 30 minutes before placing on ice. Sulfo-SDA was added at a protein to crosslinker weight ratio of 1:1 and the diazirine group of the crosslinker was photoactivated by UV irradiation at 365 nm from an CL-1000 Ultraviolet Crosslinker (Spectrum) for 20 minutes. Crosslinking of the NHS ester was allowed to proceed for a further 2 hours on ice before acetone precipitation. The protein precipitates were stored at -80°C until further processing.

#### Mass spectrometry sample processing, data acquisition, and analysis

Precipitated CLMS protein samples were resolubilized in digestion buffer (8 M urea in 100 mM ammonium bicarbonate) to an approximate protein concentration of 1 mg/ml. Dissolved protein was reduced by addition of 1 M dithiothreitol (DTT) to a final concentration of 5 mM. The reaction was incubated at room temperature for 30 minutes. Free sulfhydryl groups in the sample were then alkylated by adding 500 mM iodoacetamide to a final concentration of 15 mM and incubation at room temperature for 20 minutes in the dark. After alkylation, additional 1 M DTT was added to a total concentration of 10 mM to quench excess iodoacetamide. Next, protein samples were digested with LysC (added a at a 50:1 (m/m) protein to protease ratio) at room temperature for four hours. The sample was then diluted with 100 mM ammonium bicarbonate to reach a urea concentration of 1.5 M. Trypsin was added at a 50:1 (m/m) protein to protease ratio to further digest proteins overnight (~15 hours) at room temperature. Resulting peptides were desalted using C18 StageTips.^62^

For each sample, resulting peptides were fractionated using size exclusion chromatography in order to enrich for crosslinked peptides^63^ using a Superdex 30 Increase 3.2/300 column (Cytiva) at a flow rate of 10 μl/minute. The mobile phase consisted of 30% (v/v) acetonitrile and 0.1% trifluoroacetic acid. The earliest six peptide-containing fractions (50 ml each) were collected. Solvent was removed using a vacuum concentrator. The fractions were then analysed by LC-MS/MS.

LC-MS/MS analysis was performed using an Orbitrap Fusion Lumos Tribrid mass spectrometer (Thermo Fisher Scientific), connected to an Ultimate 3000 RSLCnano system (Dionex, Thermo Fisher Scientific). Each size exclusion chromatography (SEC) fraction was resuspended in 1.6% v/v acetonitrile 0.1% v/v formic acid and analysed with duplicated LC-MS/MS acquisitions. Peptides were injected onto a 50-centimetre EASY-Spray C18 LC column (Thermo Scientific) operated at 50°C column temperature. Mobile phase A consisted of water, 0.1% v/v formic acid and mobile phase B of 80% v/v acetonitrile and 0.1% v/v formic acid. Peptides were loaded and separated at a flowrate of 0.3 μl/min. Peptides were separated by applying a gradient ranging from 2% to 45% B over 90 minutes. Then the content of B was increased to 55% and 95% within 2.5 minutes each. Eluted peptides were ionized by an EASY-Spray source (Thermo Scientific) and introduced directly into the mass spectrometer.

MS data was acquired in data-dependent mode with the top-speed option. For each three-second acquisition cycle, the full scan mass spectrum was recorded in the Orbitrap with a resolution of 120,000. The ions with a charge state from 3+ to 7+ were isolated and fragmented using higher-energy collisional dissociation (HCD). For each isolated precursor, stepped collision energy (26%, 28% or 30%) was applied. The fragmentation spectra were then recorded in the Orbitrap with a resolution of 50,000. Dynamic exclusion was enabled with single repeat count and 60 second exclusion duration.

MS2 peak lists were generated from the raw mass spectrometric data files using the MSConvert module in ProteoWizard (v3.0.11729). Precursor and fragment m/z values were recalibrated. Identification of crosslinked peptides was carried out using xi-SEARCH software (v1.7.6.4).^64^ The peak lists were searched against the sequences, and the reversed sequences, of the 5 *S. pombe* condensin subunits. The following parameters were applied for the search: MS accuracy = 3 ppm; MS2 accuracy = 5 ppm; enzyme = trypsin (with full tryptic specificity); allowed number of missed cleavages = 3; missing monoisotopic peak = 2; cross-linker = SDA (the reaction specificity for SDA was assumed to be lysine, serine, threonine, tyrosine and protein N termini on the NHS ester and any amino acids on the diazirine side); fixed modifications = carbamidomethylation on cysteine; variable modifications = oxidation on methionine, SDA loop link, hydrolysed SDA on the diazirine end, deamidation on asparagine, acetone modification on lysine and histidine, maximum variable modifications per peptide=2.

Crosslinked peptide candidates identified by at least three matched fragment ions (at least two containing the crosslinked residue) were filtered using xiFDR.^65^ A false discovery rate of 1% on residue-pair level was applied with “boost between” option selected.

Phyre2^34^ was used to generate structural models for fission yeast condensin based on available cryo-EM structures.^17,33^ The generated structural models were uploaded to xiVIEW^64^ to measure crosslink distances and produce Circos plots. The pairs of crosslinked residues were then retrieved and visualised on the structural models using ChimeraX.^66^

#### ChIA-PET data analysis

ChIA-PET data (Sequence Read Archive: SRP061635) were retrieved and processed using Mango,^40^ as previously described.^39^ To allow Mango to evaluate inter-chromosome associations, the following modifications were applied. The average read frequency of all inter-chromosome interactions between condensin binding sites, as defined by self-ligations (average 2500 bp widths), between chromosomes A and B was used as the expected read frequency. A Poisson distribution with the expected mean read number as the λ parameter was then used to calculate a p-value for specific inter-chromosome interactions. Adjusted p-values (FDR) were calculated using the BH method.^67^ Significant intra- and inter-chromosome interactions were defined with an FDR < 0.01 and a minimum read number of two.

Circos plots were generated using the Circos software.^68^ To represent significant interactions across the genome, association anchors were extended 5 kb up- and downstream and reads from significant interactions within these windows were combined. Interactions involving telomeres and rDNA repeats (chr I coordinates: < 29,763, > 5,567,159; chr II: < 8,061, > 4,504,863; chr III < 24,810, > 2,439,540) were removed from the Circos plots due to the repetitive nature of the underlying DNA sequence. For a linear representation of intra- and inter-chromosome interactions, association anchors were extended 500 bp up- and downstream and reads from significant intra- and inter-chromosomal interactions in these windows were combined but separately counted.

#### Hi-C data analysis

Three replicates of normalized 2 kb Hi-C matrices, wild-type (wt) and Cut14 shut-off (*cut14*^*SO*^) mitotic cells, were retrieved from GEO (GSE94478).^7^ The original Hi-C scores were divided by the average of all inter-chromosome scores, resulting in an average inter-chromosome Hi-C score of 1. If all replicates from wt and *cut14*^*SO*^ cells have non-zero scores, those bin pairs were included in the analysis. Since the condensin distribution along chromosome III is obscured by the rDNA repeats at both ends, 2 kb inter-chromosome pairs from only chromosomes I and II were considered.

“ChIA-PET” sites were from above, while condensin binding sites ("Con") were from GEO accession number: GSE94478 (n=668, average size = 889 bp, median = 522 bp).^7^ The non-condensin binding sites ("Non") were generated by randomly shuffling the locations of the "Con" sites within the same chromosome, while preserving the width of the binding sites. To calculate the average Hi-C score of “ChIA-PET”, "Con" or "Non", the average Hi-C scores for the respective categories were calculated for twenty randomly selected pairs. This sampling was repeated 100 times. The statistical significance of the difference in the distributions of the 100 data points was tested using a two-sided Mann-Whitney U test.

### Quantification and Statistical Analysis

Quantitative bulk biochemical results are based on at least three independent experimental repeats. DNA and protein gels were imaged using a Chemidoc imaging system (BioRad) or Epson flatbed scanner, respectively. In gel fluorescence was recorded by an Amersham Imager 600. Band intensities were then quantified in Fiji. The individual results from all three repeats are shown, together with the means and standard deviations. In some cases, where effect sized were vast, conclusions are based on qualitative comparisons. In these cases, the experiments were also repeated at least three times and a representative example is shown. Details on quantification in the CLMS, single molecule, and ChIA-PET experiments, as well as their statistical analyses, are found in their respective Method Details sections.

## Supplementary Material

Supplementary material

## Data Availability

The protein crosslink mass spectrometry data reported in this study has been deposited with the Japan ProteOme STandard repository (identifier JPST002102) and ProteomeXchange (identifier PXD041027). The ChIA-PET data analysed in this study are available from the Sequence Read Archive with accession number SRP061635. Unprocessed and uncropped gel images have been placed in the Mendeley repository where they can be accessed at https://doi.org/10.17632/gs8j93pct8.1. The custom Python script used to analyse C-Trap data is available from Github at https://github.com/tonytang9544/CT6_C-Trap_kymograph_tracker. Any additional information required to reanalyze the data reported in this paper is available from the lead contact upon request.

## Highlights

- Bulk biochemical and single-molecule analysis of the fission yeast condensin complex
- Individual condensins engage in sequential, topological dsDNA-dsDNA capture
- A DNA-DNA capture signature emerges during *in vivo* mitotic chromosome formation
- These results support a “diffusion capture” model for chromosomal condensin function

## In brief

Tang et al. biochemically explore how the condensin complex promotes chromosome compaction during cell divisions, revealing that single condensins sequentially and topologically capture two double-stranded DNAs. The authors uncover a DNA-DNA capture signature *in vivo*, indicative of a “diffusion capture” mechanism for mitotic chromosome formation.
