# Supplementary material for: Establishment of dsDNA-dsDNA interactions by the condensin complex
Source: Mol Cell. Author manuscript; Available in PMC 2024 Feb 29. (PMC10842940; doi:10.1016/j.molcel.2023.09.019)
Supplement: Supplementary material [file EMS194253-supplement-Supplementary_material.pdf]

Molecular Cell, Volume *83*

Supplemental information

Establishment of dsDNA-dsDNA interactions

by the condensin complex

Minzhe Tang, Georgii Pobegalov, Hideki Tanizawa, Zhuo A. Chen, Juri Rappsilber, Maxim Molodtsov, Ken-ichi Noma, and Frank Uhlmann

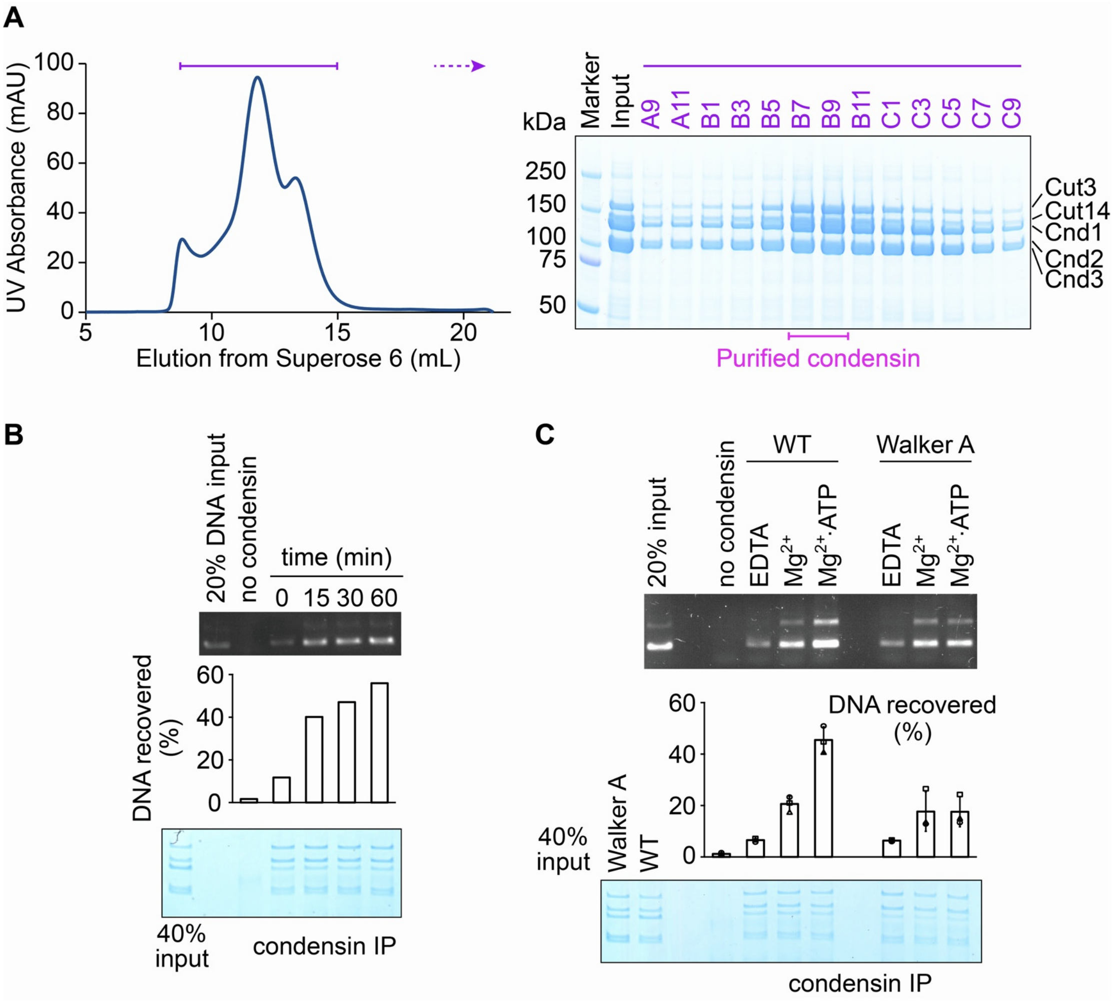

Figure S1. Biochemical characterization of fission yeast condensin, related to Figure 1

(A) Fission yeast condensin elution profile from the final gel filtration purification step. The indicated fractions (purple bar) were analysed by SDS-PAGE followed by Coomassie Blue staining. Fractions B7 - B9 (pink bar) were combined, constituting the final purified condensin preparation.

(B) Condensin loading time course experiment. Reactions were quenched at the indicated times. The recovered condensin and DNA, following high salt washes, was quantified relative to the input.

(C) Condensin loading assay comparing wild type and Walker A motif mutant condensin. The results from three independent repeat experiments are shown, boxes and whiskers indicate means and standard deviations.

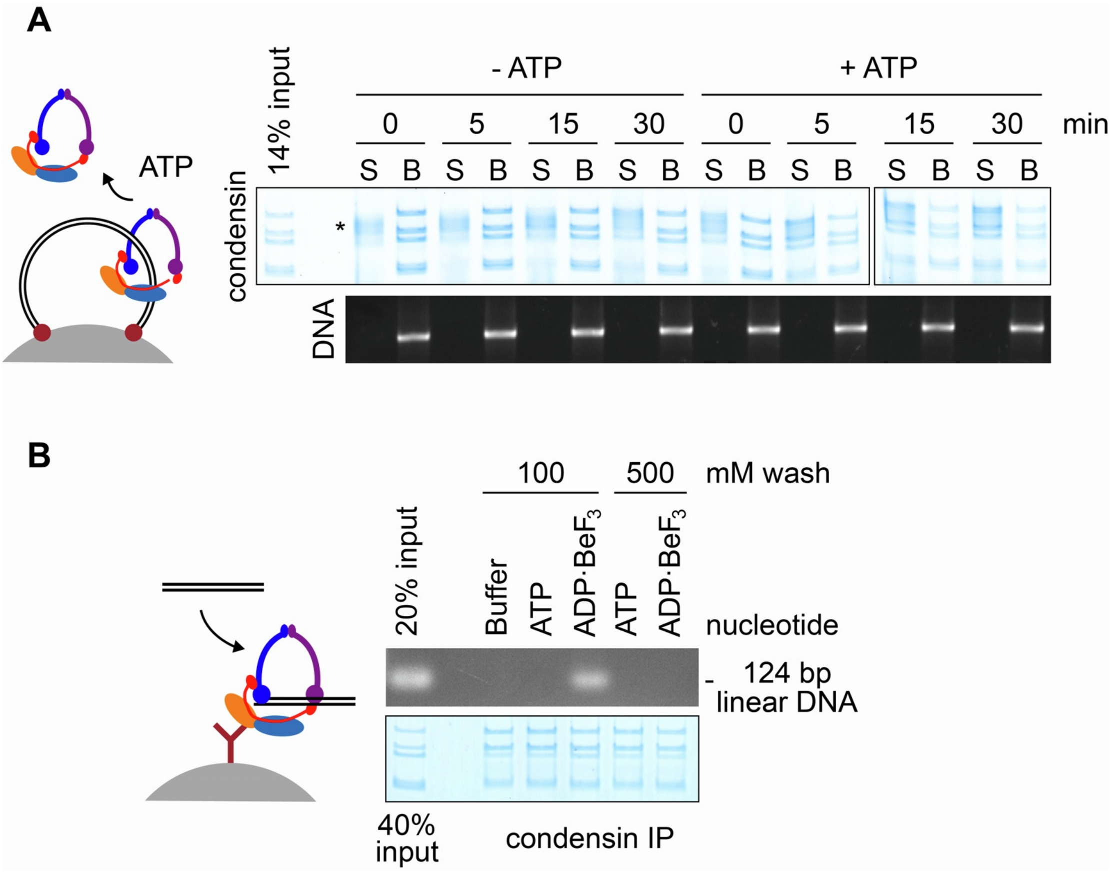

Figure S2. Reversible topological condensin loading and a condensin-DNA gripping state, related to Figure 2

(A) Time course analysis of the condensin unloading reaction. Samples at the indicated times were separated into soluble and bead-bound fractions. The asterisk indicates contaminants from BSA, part of the incubation buffer.

(B) Salt sensitivity of the condensin DNA gripping state. Condensin was incubated with a short linear dsDNA substrate. Immunoprecipitation and washes were then performed in the presence of either 100 mM or 500 mM NaCl.

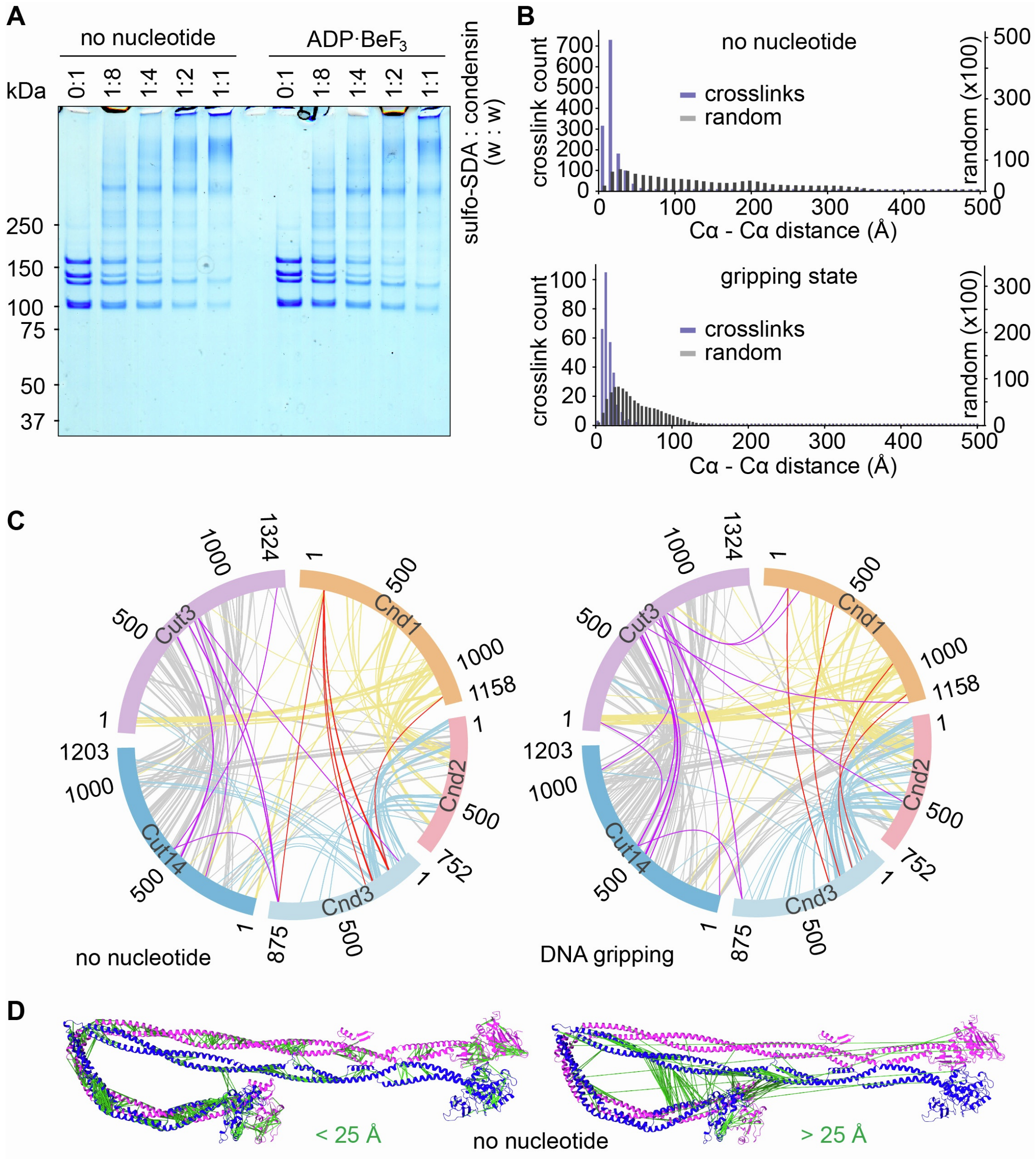

No. of crosslinks:	no nucleotide	DNA gripping
hinge - head	2	6
hinge - Cnd1	0	3
hinge - Cnd2	0	1
hinge - Cnd3	7	3

Figure S3. The condensin crosslink mass spectrometry experiment, related to Figure 3

(A) SDS-PAGE analysis of the crosslinking products of condensin-DNA complexes in the absence or presence of ADP•BeF_3, using the indicated sulfo-SDA concentrations.

(B) Histogram showing the distance distribution between α-carbons of two crosslinked residues within condensin subunits, next to the distribution of distances between randomly chosen residues, mapped onto non-engaged [S1] and gripping state [S2] structural models of fission yeast condensin, respectively.

(C) Overview of crosslinks between condensin subunits in the initial no nucleotide (left) and ADP•BeF_3-bound DNA gripping state (right). Crosslinks between the two SMC subunits are shown in grey, those involving $Cnd1^{Ycs4}$ and $Cnd3^{Ycg1}$ in yellow and blue. Crosslinks involving the SMC hinge and those between $Cnd1^{Ycs4}$ and $Cnd3^{Ycg1}$ are highlighted in purple and red, respectively.

(D) Crosslinks within the $Cut3^{Smc4}$ and $Cut14^{Smc2}$ subunits in the no nucleotide state that map onto its fission yeast structural model within reach of the sulfo-SDA crosslinker (< 25 Å) are shown on the left. Numerous crosslinks that span larger distances (> 25 Å), shown on the right, suggest substantial structural flexibility of the SMC subunits. The numbers of detected crosslinks that involve the SMC hinge and indicated parts of the condensin complex in both the no nucleotide and DNA gripping states are listed.

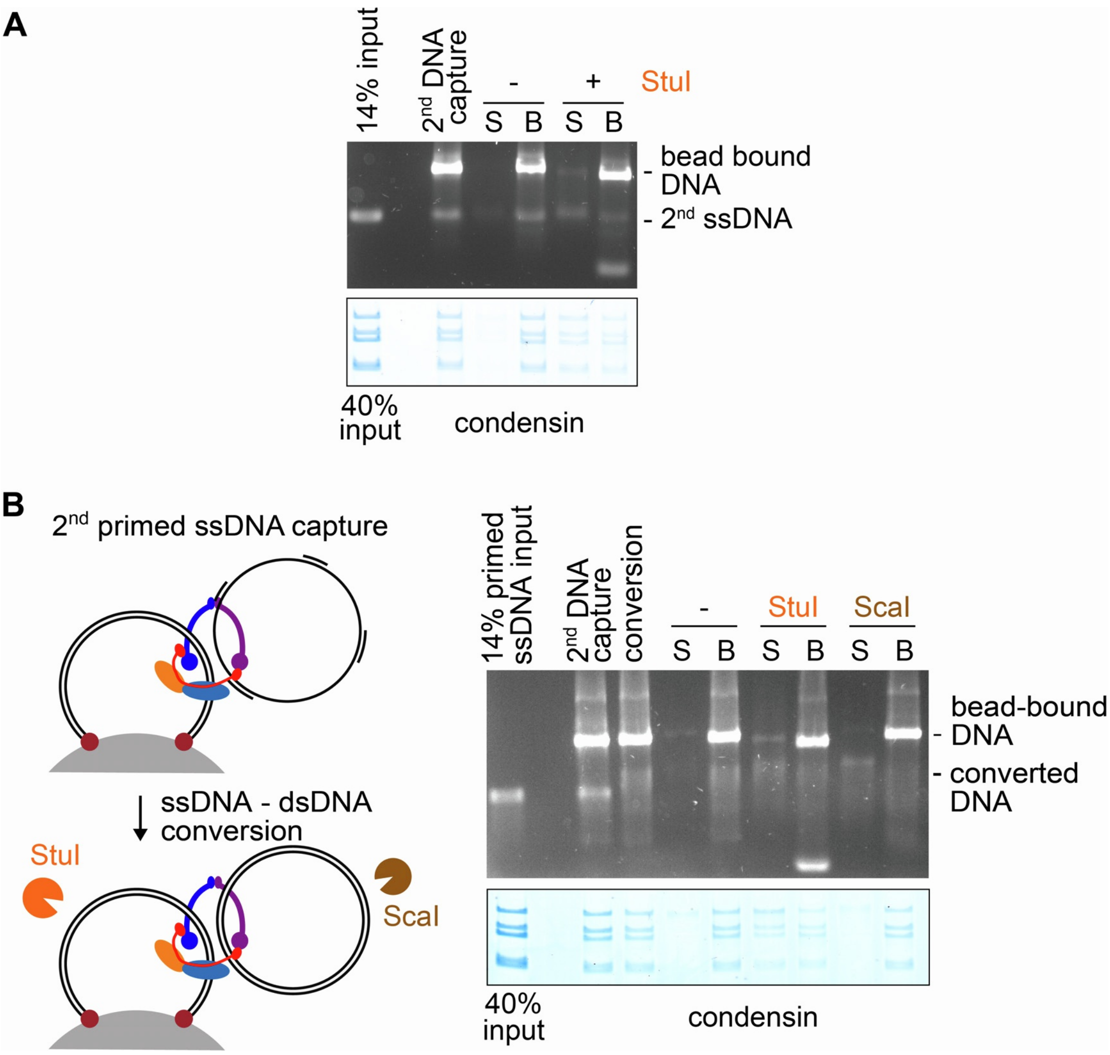

Figure S4. Characterization of second ssDNA capture, related to Figure 4

(A) The topological nature of the condensin-dsDNA association following second ssDNA capture was probed by StuI cleavage of the bead-bound dsDNA. The DNA and protein content in the soluble (S) and bead-bound (B) fractions were analysed by agarose gel electrophoresis and SDS-PAGE, respectively.

(B) The topological nature of second ssDNA capture was analysed after converting a primed ssDNA substrate to dsDNA using phage T4 DNA polymerase. Reactions were then incubated with the indicated restriction enzymes to cleave either the bead-bound or the converted second DNA.

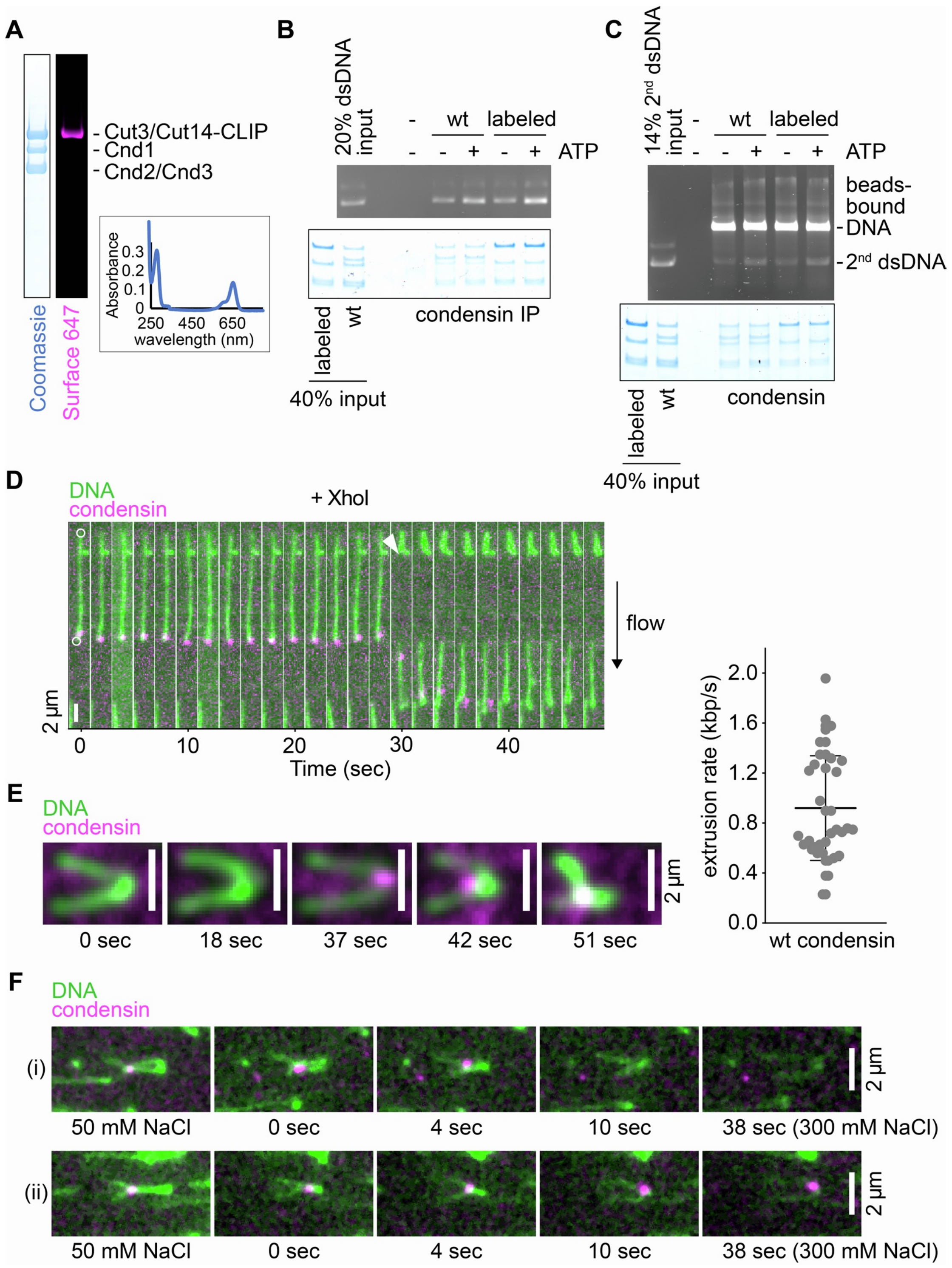

Figure S5. Characterization of single molecule imaging reagents and loop extrusion assays, related to Figure 5

(A) Purified, Surface 647-labelled Cut14-CLIP condensin was analysed by SDS-PAGE followed by Coomassie Blue staining or in-gel fluorescence imaging. An absorption spectrum of the preparation (inset) showed that 570 nM

condensin was associated with 560 nM Surface 647 dye, equating to a labelling efficiency of over 95%.

(B) Comparison of wild type and Surface 647-labelled condensin in the condensin loading assay. Following incubation with circular dsDNA in the absence or presence of added ATP, condensin was immunoprecipitated and the recovered protein and DNA were analysed by SDS-PAGE and agarose gel electrophoresis, respectively.

(C) Comparison of wild type and Surface 647-labelled condensin in the second DNA capture assay. Following loading onto bead-bound DNA, reactions were incubated with a second circular dsDNA. The protein and DNA recovered on the beads were analysed.

(D) The topological nature of condensin loading onto λ-DNA, tethered to the flow cell surface, was investigated by restriction enzyme cleavage. An image timeseries of the λ-DNA stained with SYTOX Orange (green) and condensin (magenta) is shown, while the restriction enzyme XhoI was added that cleaves the λ-DNA once. The DNA anchor points are highlighted by white circles, the DNA cleavage event by a white arrowhead, and the direction of flow by a black arrow.

(E) *In vitro* loop extrusion by fission yeast condensin. An example timeseries is shown, illustrating Surface 647-labelled condensin (magenta) forming a loop on a loosely tethered λ-DNA, stained with SYTOX Orange (green). 30 loop extrusion events out of 100 DNA molecules were observed after 7 minutes of incubation. Loop extrusion rates from an experiment using unlabelled condensin were then measured and plotted. The median and quartiles of the extrusion rate distribution are indicated.

(F) Following extrusion, loops were challenged with 300 mM NaCl-containing buffer, conditions known to resolve loops generated through loop extrusion by the cohesin complex [S3,4]. Condensin-generated loops also resolved using this treatment. In 11 out of 15 observed cases, the condensin signal was lost during loop resolution, suggesting that condensin was not topologically bound to DNA, (example (i)). In contrast, 4 out of the 15 cases showed condensin remaining stably DNA-bound (example (ii)), suggestive of topological DNA

entrapment. Note that DNA staining by SYTOX Orange is salt sensitive so that the DNA trace is more faintly visible under the elevated salt conditions. These observations suggest that loop extrusion typically, but not always, occurs without topological DNA entry into the condensin ring [S5].

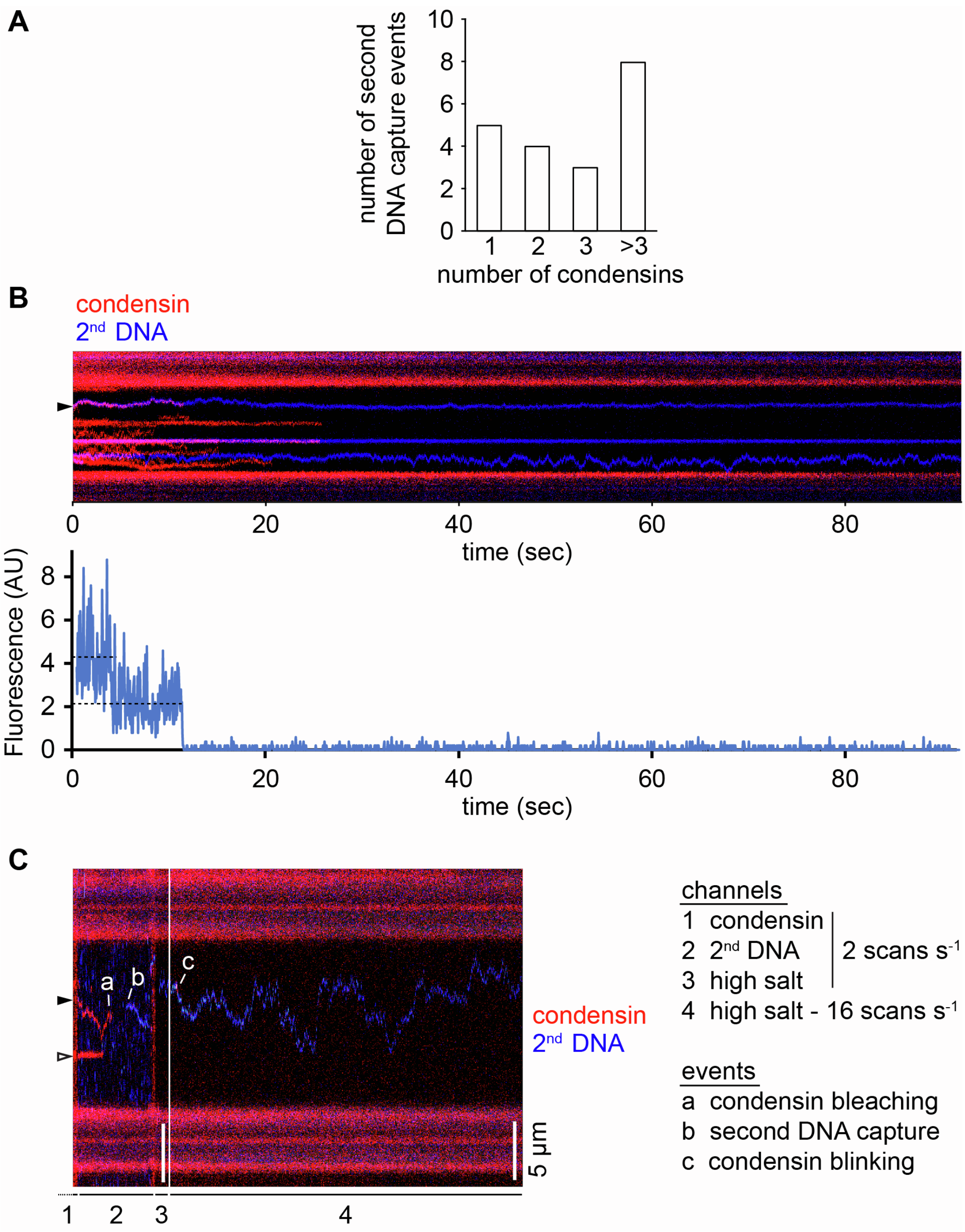

Figure S6. Second DNA capture by condensin, related to Figure 6

(A) The photobleaching steps of all condensin signals that colocalized with a second dsDNA plasmid in the experiment shown in Figure 6C were counted and plotted.

(B) Example of a condensin signal from the experiment in Figure 6D that corresponds to two condensins (highlighted by the arrowhead). Fluorescence

intensity decreased in two discrete steps, and each step corresponded to an intensity loss of similar size to single condensin bleaching steps, e.g. the step recorded in Figure 6E.

(C) Real-time observation of second dsDNA capture by condensin. Continuous imaging of the λ-DNA path, following λ-DNA stretching, in its sequentially numbered incubation channels. The solid arrowhead highlights a single condensin, identified by single-step photobleaching, and later by a brief single-step blinking event. Events are indexed a – c. The open arrowhead points to a cluster of condensins that was most likely non-topologically attached to the λ-DNA following incubation in the condensin channel, and that dissociated shortly after transfer to the 2nd DNA channel.

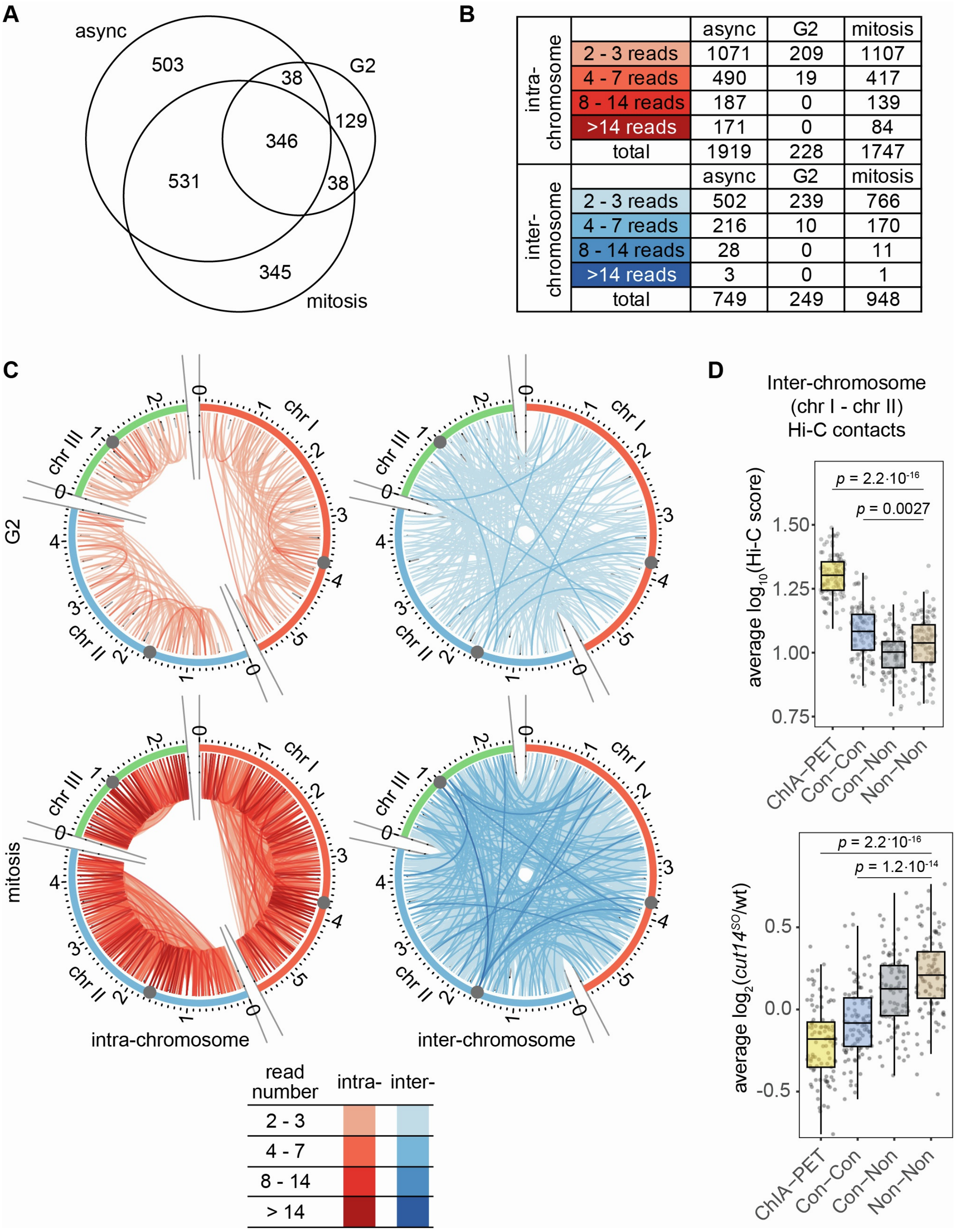

		async	G2	mitosis
intra-chromosome	2 - 3 reads	1071	209	1107
	4 - 7 reads	490	19	417
	8 - 14 reads	187	0	139
	>14 reads	171	0	84
	total	1919	228	1747
inter-chromosome	2 - 3 reads	502	239	766
	4 - 7 reads	216	10	170
	8 - 14 reads	28	0	11
	>14 reads	3	0	1
	total	749	249	948

Figure S7. Additional documentation of the condensin ChIA-PET experiment, as well as of condensin-dependent inter-chromosome interactions seen by Hi-C, related to Figure 7

(A) Venn diagram, visualising the overlap of inter-chromosome association anchors in the asynchronous (async), the G2-enriched, and the mitosis-

enriched populations (p = 1.6 x 10^{-21}, G2 vs. mitosis, two-sided Fisher's exact test).

(B) Table summarising the number of intra- and inter-chromosome associations, and their read counts, detected in asynchronous, G2-enriched and mitosis-enriched fission yeast cultures.

(C) Circos plots of condensin-linked intra- and inter-chromosome associations detected in G2- and mitosis-enriched fission yeast cultures. Colour intensities indicate read number counts. Centromeres are indicated by grey circles.

(D) Condensin-dependent inter-chromosome interactions seen by Hi-C. We analyzed inter-chromosome interactions between chromosomes I and II in a previously acquired fission yeast Hi-C dataset, comparing mitotic wild type (wt) and condensin shut-off ($cut14^{SO}$) cells [S6]. Condensin ChIA-PET positive regions (ChIA-PET) [S7], or condensin binding sites identified by ChIP-sequencing (Con) [S6], were significantly enriched for inter-chromosome interactions, when compared to condensin non-binding sites (Non) (upper panel). Following condensin shut-off, the relative interaction frequency between ChIA-PET and between Con sites decreased, while those between Non sites increased (lower panel).

Supplemental References

S1 Shaltiel, I.A., Datta, S., Lecomte, L., Hassler, M., Kschonsak, M., Bravo, S., Stober, C., Ormanns, J., Eustermann, S., and Haering, C.H. (2022). A hold-and-feed mechanism drives directional DNA loop extrusion by condensin. Science *376*, 1087-1094.

S2 Lee, B.G., Merkel, F., Allegretti, M., Hassler, M., Cawood, C., Lecomte, L., O'Reilly, F.J., Sinn, L.R., Gutierrez-Escribano, P., Kschonsak, M., et al. (2020). Cryo-EM structures of holo condensin reveal a subunit flip-flop mechanism. Nat. Struct. Mol. Biol. *27*, 743-751.

S3 Davidson, I.F., Bauer, B., Goetz, D., Tang, W., Wutz, G., and Peters, J.-M. (2019). DNA loop extrusion by human cohesin. Science *366*, 1338-1345.

S4 Kim, Y., Shi, Z., Zhang, H., Finkelstein, I.J., and Yu, H. (2019). Human cohesin compacts DNA by loop extrusion. Science *366*, 1345-1349.

S5 Higashi, T.L., and Uhlmann, F. (2022). SMC complexes: Lifting the lid on loop extrusion. Curr. Opin. Cell Biol. *74*, 13-22.

S6 Kakui, Y., Rabinowitz, A., Barry, D.J., and Uhlmann, F. (2017). Condensin-mediated remodeling of the mitotic chromatin landscape in fission yeast. Nat. Genet. *49*, 1553-1557.

S7 Kim, K.-D., Tanizawa, H., Iwasaki, O., and Noma, K. (2016). Transcription factors mediate condensin recruitment and global chromosomal organization in fission yeast. Nat. Genet. *48*, 1242-1252.
